# Computational Design, Synthesis, and Photochemistry of Cy7‐PPG, an Efficient NIR‐Activated Photolabile Protecting Group for Therapeutic Applications**

**DOI:** 10.1002/anie.202201308

**Published:** 2022-03-10

**Authors:** Georgios Alachouzos, Albert M. Schulte, Anirban Mondal, Wiktor Szymanski, Ben L. Feringa

**Affiliations:** ^1^ Centre for Systems Chemistry, Stratingh Institute for Chemistry Faculty for Science and Engineering University of Groningen Nijenborgh 4 9747 AG Groningen The Netherlands; ^2^ Department of Radiology Medical Imaging Center University Medical Center Groningen University of Groningen Hanzeplein 1 9713 GZ Groningen The Netherlands

**Keywords:** Density Functional Theory, NIR Light, Photochemistry, Photolabile Protecting Groups, Photopharmacology

## Abstract

Photolabile Protecting Groups (PPGs) are molecular tools used, for example, in photopharmacology for the activation of drugs with light, enabling spatiotemporal control over their potency. Yet, red‐shifting of PPG activation wavelengths into the NIR range, which penetrates the deepest in tissue, has often yielded inefficient or insoluble molecules, hindering the use of PPGs in the clinic. To solve this problem, we report herein a novel concept in PPG design, by transforming clinically‐applied NIR‐dyes with suitable molecular orbital configurations into new NIR‐PPGs using computational approaches. Using this method, we demonstrate how Cy7, a class of NIR dyes possessing ideal properties (NIR‐absorption, high molecular absorptivity, excellent aqueous solubility) can be successfully converted into Cy7‐PPG. We report a facile synthesis towards Cy7‐PPG from accessible precursors and confirm its excellent properties as the most redshifted oxygen‐independent NIR‐PPG to date (λ_max_=746 nm).

## Graphical abstract

The computational design and synthesis of Cy7‐PPG, a new oxygen‐independent novel near‐infrared photolabile protecting group (PPG) is reported. Cy7‐PPG is the most redshifted PPG to date (λ_max_=746 nm), with unmatched near‐infrared molecular absorptivity and aqueous solubility, and excellent uncaging efficiency, paving the way for the use of PPGs in the clinic.

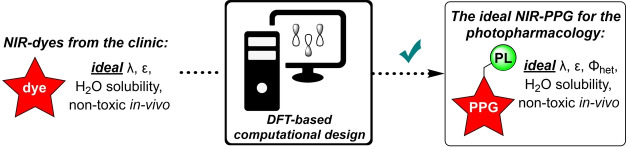

## Introduction

Photopharmacology is the scientific discipline of employing light to control therapeutic action inside a biological specimen or a living system.[^1^, ^2^, ^3^, ^4^, ^5^, ^6^, ^7^] Light is non‐invasive to the system,[^6^, ^7^, ^8^] and confers unparalleled spatiotemporal control of function, with resolutions in the order of micrometers and milliseconds.^[9]^

**Figure 1:**
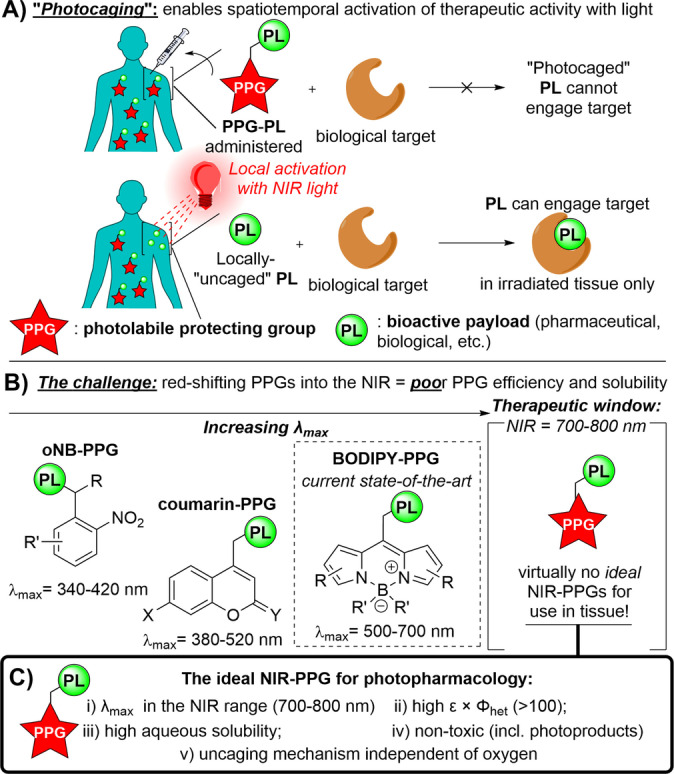
PPGs in photocaging, current PPG designs and the undiscovered ideal PPG for use in tissue.

Photolabile Protecting Groups (PPGs) are cornerstone molecular tools within photopharmacology (Figure 1a).[^10^, ^11^, ^12^, ^13^, ^14^, ^15^, ^16^, ^17^] PPGs are small molecules that are covalently bound to a bioactive payload (PL) (a small molecule drug, biological, etc.) to block (“cage”) its therapeutic action. Irradiating the PPG at its main absorption band induces the release of the PL, restoring (“uncaging”) its bioactivity.

Unsurprisingly, the extent to which PPGs can be deployed in the human body, and therefore be clinically applied, hinges heavily on the photochemical properties of the PPG (Figure 1b).[^1^, ^11^, ^13^] First, the PPG must absorb light wavelengths that are both benign to human tissue and are deep‐reaching. For this purpose, the near‐IR (NIR) wavelength range has become synonymous with the “therapeutic window”, with 700–800 nm light penetrating the deepest in human tissue.^[18]^ Beyond its absorbance, the ideal PPG must uncage efficiently (i.e. the product of its molar extinction coefficient and its uncaging quantum yield, ϵ×Φ_het_ >100 M^−1^cm^−1^),^[12]^ and be both soluble and non‐toxic to the tissue (Figure 1c). For uncaging to occur without phototoxicity and to allow for **PL** release in a wide range of biological targets, including hypoxic tissues such as solid tumors, or even bacterial biofilms, the **PL** release mechanism should be independent of the presence of oxygen.[^19^, ^20^]

Such an *ideal PPG has not yet been discovered*, and thus the advancement of PPG therapies into the clinic is currently hampered.[^11^, ^12^] Though the ideal NIR‐PPG remains elusive, tremendous recent advances have been made to “*red‐shift*” PPGs activation wavelengths from the UV or visible light range towards the NIR‐range (Figure 1b). For example, **BODIPY‐PPG** absorption bands have been red‐shifted by nearly 200 nm to generate variants now constituting the “state‐of‐the‐art” in PPGs approaching the therapeutic window.[^12^, ^21^]

However, these red‐shifting strategies employed are not without their downsides. Red‐shifting PPGs typically involves extending the conjugation of their chromophore, which results in: i) loss of PPG solubility in water due to increased lipophilicity; and ii) decreased uncaging quantum yield (Φ_het_).[^10^, ^11^, ^12^, ^22^, ^23^, ^24^] Beyond being an inherently uphill battle, red‐shifting strategies suffer from a multi‐parameter “hydra paradox” since *improvement towards one ideal property often leads to diminished progress towards another ideal property*.^[12]^

As a discipline exiting its infancy and looking to finally emerge in the clinic,[^1^, ^2^] photopharmacology *urgently needs PPGs with well‐balanced ideal properties*: i) a λ_max_ centered between 700–800 nm; ii) a high uncaging cross‐section ϵ×Φ_het_; iii) high solubility in water; iv) low (photo)toxicity; v) oxygen‐independent uncaging. Here we describe our computation‐driven development of the first NIR‐PPG to fully meet these requirements.

## Results and Discussion

**Figure 2:**
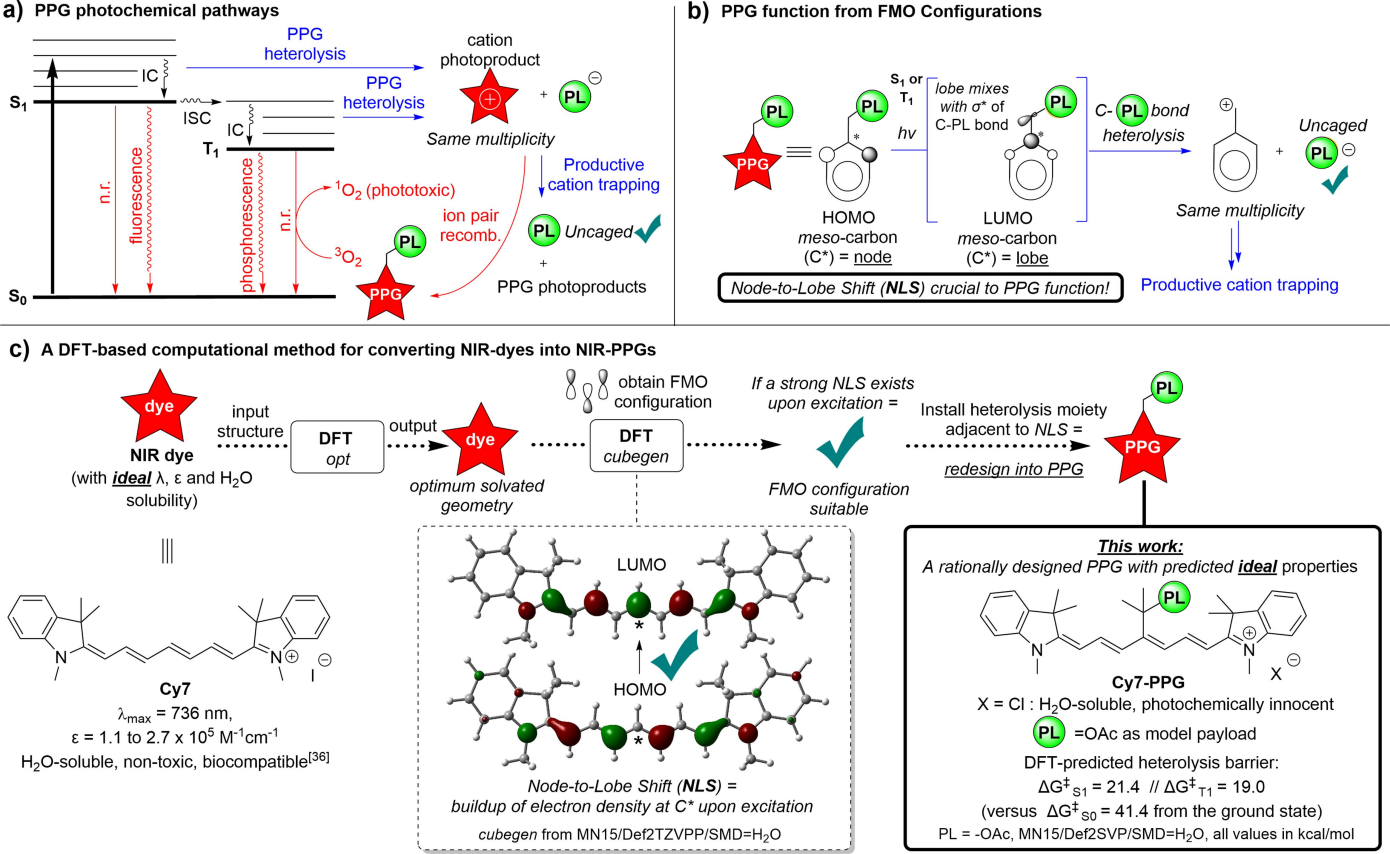
An overview of the function of PPGs and our novel DFT‐based workflow for designing ideal NIR‐PPGs.

At the outset of our quest to design a NIR‐PPG, we analyzed the fundamental photochemical pathways of existing PPGs. **PL** uncaging occurs from either the PPG excited singlet S_1_ state, or its triplet T_1_ state,^[25]^ assuming that the spin‐orbit coupled intersystem crossing (ISC) to this T_1_ state is sufficiently fast.[^10^, ^12^, ^23^] The desired heterolytic pathways are shown in blue in Figure 2a, where an ion pair of the **PL** anion and the PPG cation are formed upon heterolysis. Beyond heterolysis, various competing pathways such as radiative (e.g. fluorescence from S_1_ or phosphorescence from T_1_) or non‐radiative processes (e.g. the phototoxic sensitization of triplet oxygen to singlet oxygen from T_1_, or ion pair recombination) are analogously shown in red. Strategies toward efficient NIR‐PPGs should therefore strive to minimize the sum of all these unproductive photochemical processes.^[12]^ After **PL** heterolysis, successful interception of the PPG cation commonly takes place by the solvent. For example, nucleophilic attack by water results in an alcohol product, which unfortunately often absorbs in the same spectral range as the PPG, competing for the photons.[^10^, ^11^, ^12^, ^22^] Novel NIR‐PPGs should also disinherit this limitation.^[26]^

For our design of new NIR‐PPGs, we also considered that the heterolytic function of a PPG hinges on the changes of the frontier molecular orbital (FMO) configurations upon excitation by light,[^10^, ^12^] similar to those observed in the photoheterolysis of phenol derivatives (known as the Zimmerman *meta effect*).[^27^, ^28^] In the ground state, the populated HOMO of a PPG must possess no orbital coefficient (*an orbital node*) at the *meso‐*carbon (indicated by an * in Figure 2b), thus positioning no electron density *β* to the leaving group **PL**. The ground state FMO configuration thus prevents heterolysis in the absence of irradiation. Conversely, excitation of the PPG chromophore to either the S_1_ (or T_1_ state after ISC) populates the LUMO, which now possesses a high orbital coefficient (*an orbital lobe*) at the *meso*‐carbon. This excited state FMO configuration thus positions a high electron density *β* to the leaving group **PL**. Consequently, this newly populated LUMO mixes with the σ*‐orbital of the C‐**PL** *σ*‐bond, which results in the breaking of the bond and ultimately drives heterolysis of **PL**.

We have considered this FMO *Node‐to‐Lobe Shift* (NLS) as a crucial functional element for our forward engineering of the ideal NIR‐PPG.^[28]^ We hypothesized that existing NIR‐dye scaffolds that absorb a sufficient amount of light at an appropriate wavelength (namely, 700–800 nm) and have a well‐defined NLS in their FMO configurations could be engineered into PPGs by inclusion of a key functional moiety: a **PL** leaving group *β* to their NLS. Since the introduction of this moiety is a minimal molecular modification, one would expect the properties (λ_max_, ϵ, aqueous solubility, biocompatibility etc.) of the starting NIR‐dye to be largely conserved in the engineered NIR‐PPG. This design principle liberates us from starting our search for ideal PPG among the typical PPG scaffolds (Figure 1) and instead allows us to engineer PPG functionality into NIR‐dyes already possessing ideal properties. To the best of our knowledge there are no reported cases of the NLS being deliberately utilized towards the development of NIR‐PPGs from NIR‐dyes, suggesting that this FMO approach towards NIR‐PPGs is unexploited.

This design approach culminated in a computational workflow (Figure 2) for obtaining the FMO configurations from the DFT‐optimized structures of potential NIR dye candidates with *ideal properties* (λ_max_, ϵ, aqueous solubility, biocompatibility etc.). With this method, the candidate dyes’ FMO configurations are extracted from the DFT wavefunctions obtained at the MN15 / Def2TZVPP / SMD=H_2_O level (see Supporting Information for further computational details). The combination of global‐hybrid exchange‐correlation functional with the minimally‐augmented triple‐ζ basis set and the Solvation Model based on Density (SMD) method was expected to give a good balance of predictive accuracy versus computational cost.[^29^, ^30^, ^31^] Thereafter, the dye FMO configurations can be conveniently screened for the existence of an *NLS*. Indeed, we demonstrate here that if a strong *NLS* is present in a NIR‐dye FMO configuration, the dye can be redesigned into a PPG.

One such NIR‐dye that was examined was **Cy7**, a blockbuster fluorescent heptamethine dye used extensively in biology (Figure 2c).[^32^, ^33^] **Cy7** has a λ_max_ of 736 nm, precisely in the therapeutic window, and also exhibits a molecular absorptivity ϵ of the order of 10^5^ M^−1^cm^−1^ in aqueous media.[^34^, ^35^, ^36^] Furthermore, **Cy7** and the broader family of heptamethine dyes have extensive clinical applications, such as NIR‐imaging and photodynamic therapy.[^32^, ^37^] As a result, heptamethine dyes and their photoproducts have been well studied in terms of their in vivo effects, and are generally regarded to be safe for use in humans.^[32]^ In sum, **Cy7** is a privileged starting point for the design of *ideal PPGs* for use in the clinic.

In fact, heptamethine chromophores have been successfully incorporated into photorelease systems that do not proceed via simple heterolytic step, as the PPGs previously discussed (Figure 2b*)*.[^38^, ^39^] In these seminal systems, the photorelease of **PL**s was achieved by harnessing the oxygen photosensitizer properties of heptamethine dyes (Φ_P.S._ <0.5 %) excited by 690 nm light. Unfortunately, a downside is that these systems require singlet oxygen for its release mechanism, and thus **PL** release is not uncoupled from phototoxicity. This also prohibits the applications of these photocaged systems in hypoxic tissues.[^19^, ^20^] Lastly, the λ_max_ of this photorelease system is centered outside the ideal wavelength range of 700–800 nm.^[18]^ Thus, the *ideal PPG* based on heptamethine dyes such as **Cy7** is yet undiscovered.

Employing the computational workflow shown in Figure 2, **Cy7** was indeed found to have a strong *NLS* at its *meso*‐carbon. In our redesign of **Cy7** into a PPG, we first elected to introduce a 2‐substituted propanol subunit as the heterolytic moiety, positioning a β‐leaving group adjacent to the *NLS*. We hypothesized that the 2‐substituted propanol subunit would confer stability to the heptamethine PPG design since the central carbon is fully substituted and is thus non‐enolizable. Next, we considered the heptamethine cation counterion. **Cy7** as the iodide salt already enjoys superior solubility in water compared to many fluorescent dyes.^[36]^ Nonetheless, we instead sought to develop this PPG as the chloride salt, as chloride is photochemically innocent and should also further increase the PPG's water solubility versus iodide.[^40^, ^41^, ^42^] These combined design elements allowed us to redesign NIR‐dye **Cy7 i**nto **Cy7‐PPG** (Figure 2).

An additional support for our PPG design came from the computed thermochemistry of heterolysis from the TD‐DFT excited states of **Cy7‐PPG**. At the TD‐MN15/Def2SVP/ SMD=H_2_O level, with PL=AcOH (Figure 2c) as the archetypal model PPG leaving group,[^21^, ^43^] a modest barrier for heterolysis from the excited S_1_ state (Δ*G* ^≠^_S1_=21.4 kcal mol^−1^) was found. Furthermore, since the parent dye **Cy7** undergoes rapid ISC to its low lying triplet T_1_ state,[^34^, ^35^, ^36^] the thermochemistry of heterolysis of **Cy7‐PPG** from T_1_ was computed as well. We were pleased to find an even lower barrier of Δ*G* ^≠^_T1_=19.0 kcal mol^−1^ for the T_1_ heterolysis. Gratifyingly, the analogous computed heterolysis barrier from the ground state S_0_ was found to be prohibitively high (Δ*G* ^≠^_S0_=41.4 kcal mol^−1^), suggesting **Cy7‐PPG** should be a stable molecule in the ground state. Overall, we concluded that widely employed **PL**s (e.g. carboxylates)[^10^, ^13^, ^21^, ^22^, ^23^, ^24^, ^25^, ^43^] should be heterolyzed from **Cy7‐PPG** upon irradiation, especially if the effective heterolysis barriers were even lower due to higher vibrational states being accessed upon excitation.

**Scheme 1:**
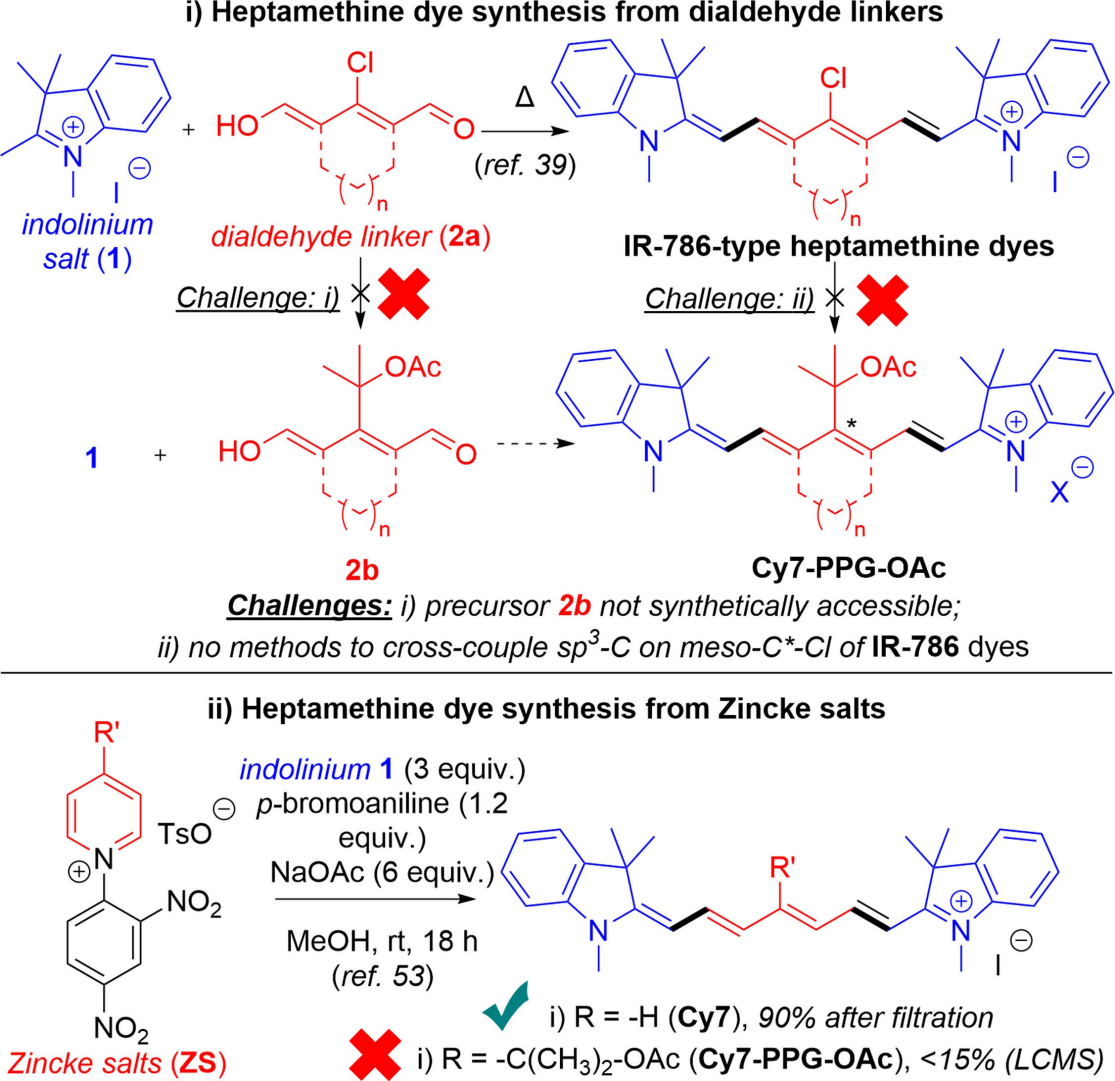
Attempted synthetic approaches to **Cy7‐PPG**.

We thus set out to develop a synthesis towards **Cy7‐PPG**, with **PL**=OAc, the archetypal **PL** used throughout previous studies,[^10^, ^13^, ^21^, ^22^, ^23^, ^24^, ^25^, ^43^] and also used in our computational evaluation (Figure 2c). Heptamethine dyes (e.g. **IR‐786**) are classically formed via double condensation of tetramethylindolinium salts with conjugated dialdehyde reactant partners.^[44]^ In these cases, the synthesis of **IR‐786** derivatives with a *meso‐*chloro substituent are facile.^[45]^ From here on, functionalizing the *meso‐carbon* with various nucleophiles has been demonstrated.[^33^, ^45^, ^46^, ^47^, ^48^] Furthermore, the Suzuki cross‐coupling of **IR‐786** derivatives has also been accomplished,[^49^, ^50^, ^51^] despite the low reactivity of the C−Cl bond (Scheme 1).

However, the synthesis of heptamethine dyes with bulky *sp* ^*3*^‐carbon *meso‐*substituents, such as our target molecule **Cy7‐PPG**, remains challenging (Scheme 1). Unfortunately, our efforts to synthesize a suitable dialdehyde variant **2** **b** with a fully substituted geminal dimethyl *sp* ^*3*^‐carbon attached to the *meso‐*carbon were unsuccessful. Our attempts to effect cross‐coupling of **IR‐786** variants towards **Cy7‐PPG** were also unsuccessful, prompting us to explore other synthetic approaches towards **Cy7‐PPG**.

**Scheme 2:**
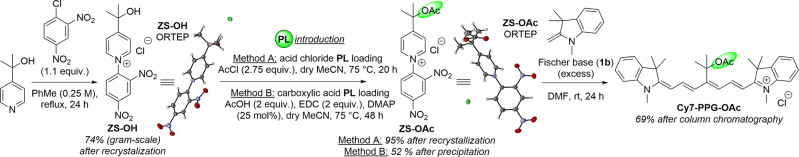
An efficient synthesis toward **Cy7‐PPG**, with two methods for loading **ZS‐OH** with a payload.

As such we turned to an alternative synthetic strategy involving activated Zincke pyridinium salts as synthetic precursors.^[52]^ Zincke salts have been successfully deployed as masked conjugated dialdehydes, able to react in the presence of aniline promoters, with carbon nucleophiles such as indolinium **1** to ultimately form heptamethine systems.^[53]^ Using this method, we successfully synthesized the parent dye **Cy7** from Zincke salt **ZS** in excellent yield (Scheme 1). However, the method was found to perform poorly towards the desired target **Cy7‐PPG‐OAc**. The low yield shown in Scheme 1 was obtained regardless of whether the reaction was stopped at low or high conversion and was determined to be due to decomposition of the product **Cy7‐PPG** by the combination of reactants, promoters, and solvent in this reaction mixture. The poor performance of this reaction is supported by the lack of synthetic examples of **Cy7** variants with bulkier or electron‐donating groups on the *meso‐*position from the published scope of this method,^[53]^ but the reason for this incompatibility is not clear. Furthermore, this reaction yielded **Cy7‐PPG‐OAc** as the iodide salt, and not the desired chloride salt. Thus, we optimized the reaction to develop a reliable, high yielding and facile synthesis of **Cy7‐PPG‐OAc** with the desired chloride counterion, (Scheme 2 *)*.

The required **ZS‐OAc** chloride salt can be conveniently accessed in two steps, with no extensive purification needed. First, commercially available 2‐(4‐pyridyl)‐2‐propanol underwent S_N_Ar reaction with commercially available 2,4‐dinitrobenzene, to furnish gram‐scale amounts of **ZS‐OH** in 74 % yield as X‐ray quality crystals after a single recrystallization. At this stage it was hypothesized that **ZS‐OH** could be loaded with either an acid chloride or a carboxylic acid to serve as the **PL** for the photochemical evaluation. Indeed, even in the absence of a base, the bulky alcohol **ZS‐OH** underwent smooth esterification in dry MeCN with acetyl chloride to yield **ZS‐OAc** chloride salt in 95 % yield, also as X‐ray quality crystals after a single‐pass recrystallization. Likewise, a second method involving a Steglich esterification with AcOH promoted by EDC also furnished the same salt **ZS‐OAc**,^[54]^ in more moderate yield.

Following the development of the **ZS‐OAc** synthesis, we improved upon the ring‐opening reaction of **ZS‐OAc** (Scheme 1) by switching the solvent to non‐protic DMF, by excluding all reaction promoters and by exchanging the indolinium salt **1** for its Fischer free base **1** **b**. These conditions furnish the desired **Cy7‐PPG‐OAc** as the chloride salt in 69 % yield.

**Figure 3:**
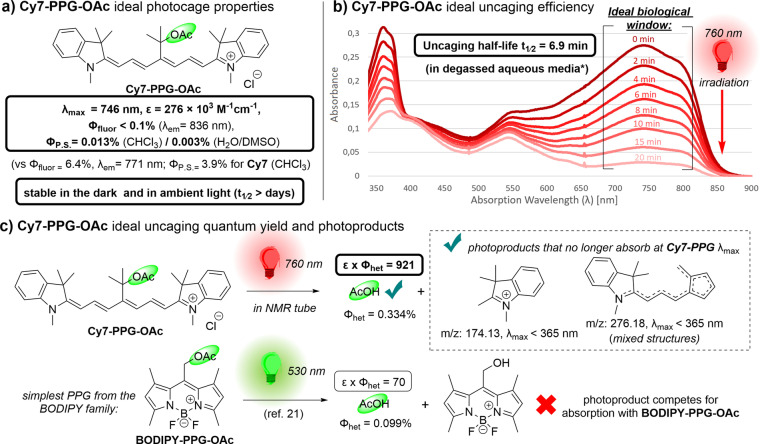
**Cy7‐PPG‐OAc** ideal photocage properties and payload uncaging with 760 nm light. a) Ideal wavelength λ_max_, molecular absorptivity ϵ, low fluorescence and singlet oxygen photosensitization quantum yields Φ_fluor_ and Φ_P.S._, and high stability of **Cy7‐PPG‐OAc**. b) UV/Vis monitored uncaging of **Cy7‐PPG‐OAc** upon irradiation with 760 nm light (*1 μM in degassed 99 : 1 milli‐Q H_2_O/DMSO). c) NMR monitored uncaging of **Cy7‐PPG‐OAc** and comparison of its uncaging cross‐section ϵ×Φ_het_ to **BODIPY‐PPG‐OAc** (see Supporting Information).

Spectroscopic evaluation of **Cy7‐PPG‐OAc** revealed its ideal properties as a photocaged compound (Figure 3): we pleasingly found **Cy7‐PPG‐OAc** to absorb at a λ_max_ of 746 nm, with a high ϵ of 2.76×10^5^ M^−1^cm^−1^ in aqueous solvent with good solubility, and it proved to be stable at pharmacological concentrations versus glutathione, cysteine and toward human plasma‐like media (see Supporting Information). We also found that **Cy7‐PPG‐OAc** is stable in solution at room temperature for days in the dark and under ambient light. We then explored other key photochemical properties of **Cy7‐PPG‐OAc**. The parent dye **Cy7** is moderately fluorescent (Φ_fluor_=6.4 %, λ_em_=771 nm),^[36]^ yet in sharp contrast, **Cy7‐PPG** was found to be very weakly fluorescent (Φ_fluor_ <0.1 %, λ_em_=836 nm). Dye **Cy7** is also a moderate oxygen photosensitizer (Φ_P.S._=3.9 %) by virtue of its ISC to its T_1_ state.[^36^, ^55^] However, in a direct comparison to its parent dye **Cy7**, **Cy7‐PPG‐OAc** was found to generate far less singlet oxygen upon excitation, with a Φ_P.S._ of only 0.013 % (CHCl_3_) or 0.003 % (H_2_O/DMSO) (see SI). Indeed, similar phenomena have been observed for related dyes, where engineering a more sterically crowded heptamethine chain results in a lower the Φ_P.S._ from the excited state of the dye, perhaps by shielding the chromophore from triplet‐triplet annihilation with ambient molecular oxygen.^[56]^ Alternatively, the bulky geminal dimethyl moiety in **Cy7‐PPG** may be enabling faster, non‐radiative return to the ground state via the “loose bolt” effect.^[57]^

Encouraged by all these gratifying observations, we proceeded to investigate the uncaging of **Cy7‐PPG**. To our delight, and fully vindicating our extensive design efforts, we observed that upon irradiation with 760 nm light, **Cy7‐PPG‐OAc** rapidly uncaged our model **PL** AcOH in a matter of minutes in degassed aqueous media (Figure 3b). We determined the heterolysis cross‐section ϵ×Φ_het_ for the uncaging of this moderate leaving group to be 921 M^−1^cm^−1^, more than an order of magnitude higher than the ϵ×Φ_het_ of the same **PL** from **BODIPY‐PPG‐OAc** (see Supporting Information).^[21]^ Interestingly, aside from successfully uncaged **PL**, an unexpected mixture of photoproducts derived from the PPG was observed during the photoheterolysis shown in Figure 3c. LCMS showed that after uncaging of AcOH, the chromophore fragmented into two unsymmetrical parts which had no absorption in the NIR wavelength range (a proposed cationic rearrangement mechanism is shown in the Supporting Information).^[58]^ Ergo, the photolysis of **Cy7‐PPG‐OAc** does not generate photoproducts that compete for photons required for the PPG's activation, as is the case for other **PPGs**.[^10^, ^11^, ^12^, ^22^, ^23^, ^24^]

Notably, the NIR light‐induced **PL** release for our designed **Cy7‐PPG‐OAc** does not require oxygen, as evidenced by: i) our quantum yield of uncaging Φ_het_=0.334 % (Figure 3c) which is two orders of magnitude higher than the oxygen photosensitization quantum yield Φ_P.S._=0.003 % in similar solvent (Figure 3a); and ii) the fact that **PL** uncaging occurs smoothly in degassed aqueous media (Figure 3b). This observation is further supported by our predicted computational hypothesis about the **PL** heterolytic step (Figure 2c). This is especially true when considering the large observed stokes shift (90 nm, Figure 3a), showing that higher S_1_ (and potentially T_1_) vibrational states are accessed upon excitation, lowering the effective barriers for **PL** heterolysis upon excitation.

**Figure 4:**
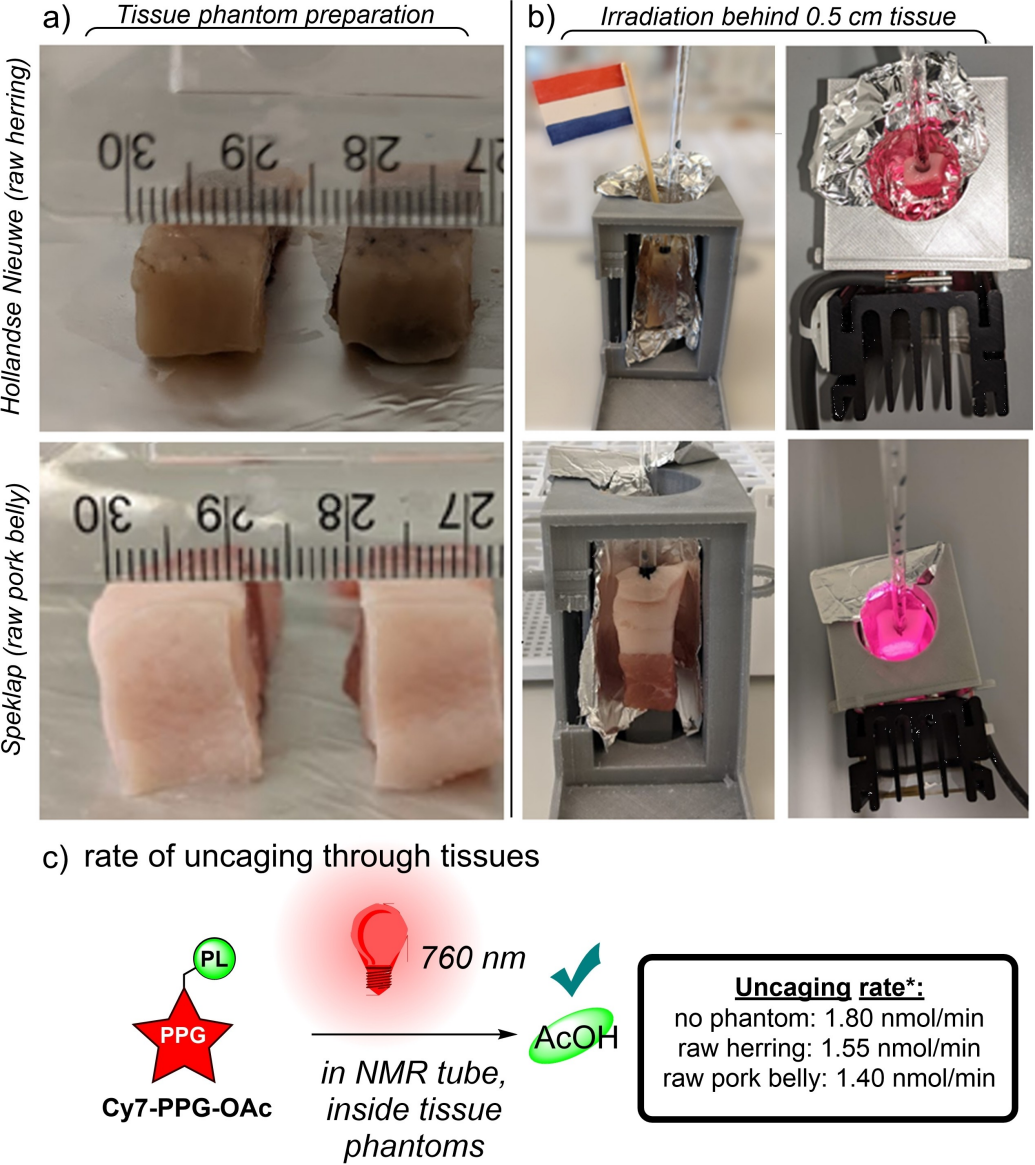
**Cy7‐PPG‐OAc** uncaging with 760 nm light inside of tissue phantoms. a) Preparation of 1 cm cuboid tissue phantoms of *Hollandse Nieuwe* (raw herring) and *Speklap* (raw pork belly). b) Mounted tissue phantoms containing a sample of **Cy7‐PPG‐OAc** (0.5 ml, 2 mM in 1 : 1 D_2_O/*d_6_‐*DMSO). c) Rate of **PL** uncaging upon 760 nm irradiation through the tissue phantoms (*given in nmol/min).

Finally, with **Cy7‐PPG** in hand, we sought to demonstrate our ability to uncage the **Cy7‐PPG‐OAc** within complex tissue phantoms (Figure 4). To this end, samples of **Cy7‐PPG‐OAc** were irradiated with 760 nm light inside a series of a readily available Dutch food samples, namely *Hollandse Nieuwe* (raw Dutch herring) and *Speklap* (raw pork belly). Unsurprisingly, 760 nm light passes unfettered through roughly 0.5 cm of these tissues to induce heterolysis of AcOH from **Cy7‐PPG‐OAc**, with little impact on photolytic rate: 86 % and 78 % uncaging rate for the fish and porcine tissue, respectively, versus 760 nm irradiation without a tissue phantom.

## Conclusion

In summary, we reported here the computational design of a new NIR‐triggered PPG with *ideal properties* for use in biomedical applications. We demonstrate that **Cy7‐PPG** is efficiently synthesized from commercial precursors, and we show that **PL**s can be readily loaded using two different synthetic methods.

**Cy7‐PPG** is the most red‐shifted (λ_max_=746 nm, ϵ = 276 × 10^3^ M^−1^cm^−1^ heterolytic PPG reported to date.[^12^, ^21^, ^23^, ^43^] The overall efficiency (ϵ×Φ_het_=921 M^−1^cm^−1^ at 746 nm) of **Cy7‐PPG‐OAc** is within the ideal range (>100 M^−1^cm^−1^),^[12]^ and is an order of magnitude higher than that of the “base‐model” of “state‐of‐the‐art” **BODIPY‐PPG**s.[^12^, ^13^, ^21^, ^23^, ^43^] **Cy7‐PPG** is also soluble in aqueous media at pharmacological concentrations. Furthermore our redesign of **Cy7** into **Cy7‐PPG** has ablated two photochemical pathways that are seen as less‐than‐desirable in PPGs: i) fluorescence, which competes with payload heterolysis; and ii) oxygen sensitization, a process that results in PPG phototoxicity.^[12]^ **Cy7‐PPG** is thus a NIR‐photocage system whose release mechanism is entirely uncoupled from singlet oxygen phototoxicity. Finally, we have demonstrated that **Cy7‐PPG** can be activated within model complex animal tissues using NIR light.

This advancement in NIR‐PPG technology paves the way towards clinical application of light for local activation of therapeutic effects in deep tissue, with unprecedented spatial and temporal precision.

## Experimental Section

Full synthetic methods, analytical and photochemical data, and computational data are provided as Supporting Information.

**Note added in proof**: After the initial submission of this manuscript for peer review, a study by Stacko and co‐workers was posted on the preprint server ChemRxiv,^[59]^ featuring Cy7 as a starting point for the development of PPGs using mostly a secondary carbon as the attachment point for the leaving group.

## Conflict of interest

The authors declare no conflict of interest.

1

## Supporting information

As a service to our authors and readers, this journal provides supporting information supplied by the authors. Such materials are peer reviewed and may be re‐organized for online delivery, but are not copy‐edited or typeset. Technical support issues arising from supporting information (other than missing files) should be addressed to the authors.

Supporting Information

Supporting Information

Supporting Information

## Data Availability

The data that support the findings of this study are available in the Supporting Information of this article.
