# Supplementary material for: Computational Design, Synthesis, and Photochemistry of Cy7‐PPG, an Efficient NIR‐Activated Photolabile Protecting Group for Therapeutic Applications
Source: Angew Chem Int Ed Engl. 2022 Mar 10;61(27):e202201308. doi: 10.1002/anie.202201308 (PMC9311213; doi:10.1002/anie.202201308)
Supplement: Supplementary file 1 — Supporting Information [file ANIE-61-0-s001.pdf]

Angewandte Chemie
Eine Zeitschrift der Gesellschaft Deutscher Chemiker

Supporting Information

Computational Design, Synthesis, and Photochemistry of Cy7-PPG, an Efficient NIR-Activated Photolabile Protecting Group for Therapeutic Applications

G. Alachouzos, A. M. Schulte, A. Mondal, W. Szymanski*, B. L. Feringa**

Supporting Information

Contents

1. Computational Methods 2
   1.1 Overview of Methods and Results 2
   1.2 Optimized Geometries and XYZ Coordinates 4
2. Synthetic Methods 25
   2.1 General remarks 25
   2.2 Synthetic Methods[4] 26
   2.3 NMR Spectra 28
   2.4 Cy7-PPG HRMS Spectrum 32
   2.5 X-ray Crystal Structures 33
3. Photochemical Methods 36
   3.1 **Cy7-PPG-OAc** Photophysical Properties, Aqueous Solubility and Stability 36
   3.2 **Cy7-PPG-OAc** Fluorescence 37
   3.4 Photoheterolysis of **Cy7-PPG-OAc** 40
   3.6 Photoheterolysis of **Cy7-PPG-OAc** in Tissue 43
4. References 44

1. Computational Methods

1.1 Overview of Methods and Results

All computational input files were prepared in GaussView 6.0 on a local Windows 10 terminal. Input files were then transferred to the Rijksuniversiteit Groningen Peregrine HPC cluster where DFT or TD-DFT calculations were carried out using the Gaussian 16 (g16) suite of programs.

To screen potential NIR-dye candidates for a *Node-to-Lobe Shift* (NLS) their frontier molecular orbital (FMO) configurations were first visualized from DFT-optimized structures. FMO visualizations of NIR-dye **Cy7** were extracted from cube files containing atom density and position data of the ground state geometry previously optimized at the MN15 functional and Def2TZVPP basis set level of theory with implicit solvation using the Solvation Model based on Density (SMD = water).[1–3] Cube files for the HOMO and LUMO of **Cy7** were generated from the g16 *cubegen* utility from checkpoint files of the previously completed optimizations.

The *meso*-carbon (*) of the DFT-optimized **Cy7** chromophore shows a clear *NLS*. Thus a 2-propanol photoheterolysis group was incorporated to transform **Cy7** into **Cy7-PPG** (*Figure S1*).

Figure S1. A DFT-based Workflow for Converting Therapeutically Suitable *Ideal* Dyes into *Ideal* PPGs.

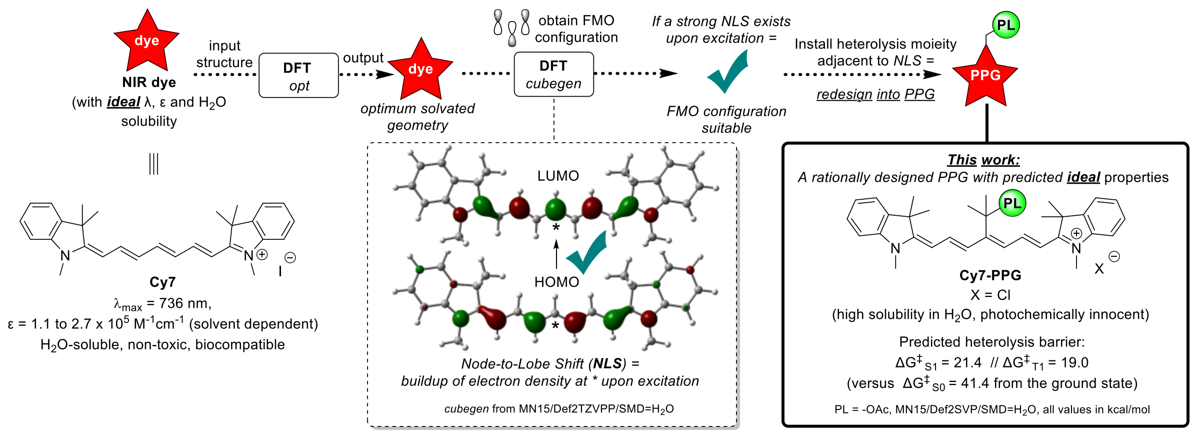

The DFT thermochemistry of heterolysis for **Cy7-PPG** was examined before its synthesis. Geometry optimization of **Cy7-PPG** to S_0, S_1 or T_1 minima or heterolysis transition states was carried out using the g16 *opt* command at the MN15 functional and Def2SVP basis set level of theory with implicit solvation using the Solvation Model based on Density (SMD = water).[1–3] Transition state geometry inputs were the result of rational guess based on bond-breaking atomic distances, or were the result of potential energy surface relaxed coordinate scans using the g16 *scan* command at the MN15/Def2SVP/SMD=water level. Intrinsic reaction coordinate (IRC)iv calculations were carried out on the transition state structures to verify that they connected to the associated reactant and product minima structures.

Figure S2. Cy7-PPG thermochemistry of heterolysis for the S0, S1 or T1 states

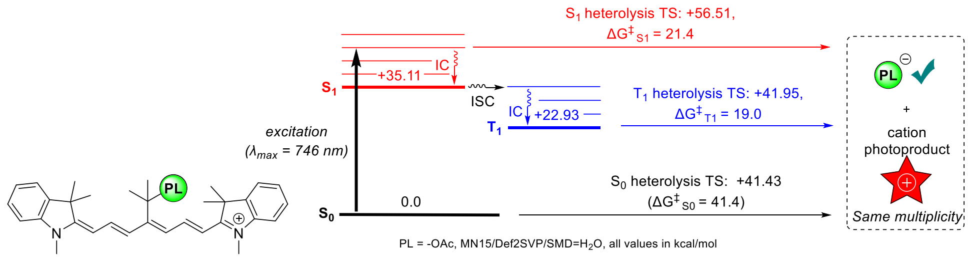

After optimization, frequency DFT calculations of all obtained optimized structures were carried out using the g16 *freq* command at the MN15/Def2SVP/SMD=water level, to confirm that ground state structures had zero imaginary frequencies and that transition states had a single imaginary frequency. All shown free energies (*Figure S2*) are ZPE and thermally corrected and were obtained from the frequency calculations. All shown free energies are reported in kcal/mol, at 298.15 K and 1 atm.

1.2 Optimized Geometries and XYZ Coordinates

Cy7 optimized geometry (# opt scrf=(smd,solvent=water) def2tzvpp mn15)

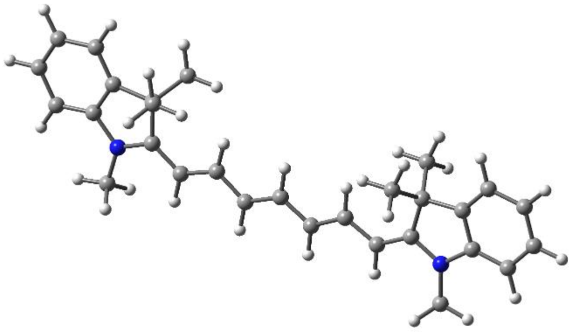

1 1

H	0.00000000	-0.17833900	0.00051200
C	0.00000000	0.91015300	0.00048300
C	-1.23858400	1.54301400	0.00089500
C	1.23858400	1.54301400	-0.00000200
C	-2.41595600	0.80795800	0.00096200
H	-1.28602200	2.62786500	0.00106000
C	2.41595600	0.80795900	-0.00016400
H	1.28602200	2.62786500	-0.00023900
C	-3.69312600	1.36587200	0.00141900
H	-2.30724300	-0.27101800	0.00063300
C	3.69312500	1.36587200	-0.00084400
H	2.30724400	-0.27101700	0.00024300
C	-4.87380000	0.63779400	0.00084200
H	-3.76544000	2.44773700	0.00215800
C	4.87380000	0.63779500	-0.00054300
H	3.76544000	2.44773700	-0.00161500
C	-5.06623000	-0.87205100	-0.00105300
N	-6.07958300	1.23860900	0.00161300
C	5.06623000	-0.87205000	0.00116300
N	6.07958200	1.23860900	-0.00154400
C	-4.49754600	-1.53013300	1.26094700
C	-4.49811400	-1.52637100	-1.26527800
C	-6.57066100	-0.97683700	-0.00092000
C	-6.26893300	2.67526400	0.00318600
C	-7.12440200	0.29724500	0.00057600
C	4.49721700	-1.52998500	-1.26076800
C	4.49844300	-1.52651800	1.26545700
C	6.57066100	-0.97683700	0.00062800

C	6.26893300	2.67526400	-0.00301800
C	7.12440200	0.29724500	-0.00090200
H	-4.88371400	-1.04681400	2.15882500
H	-4.80149400	-2.57822300	1.27309600
H	-3.40929200	-1.48984300	1.28057100
H	-4.88532100	-1.04088100	-2.16154000
H	-4.80134100	-2.57463900	-1.28003200
H	-3.40990400	-1.48516700	-1.28564100
C	-7.38362800	-2.09204500	-0.00209700
H	-5.81563700	3.11558700	0.89063700
H	-5.81606300	3.11747400	-0.88354500
H	-7.33082800	2.89661100	0.00367900
C	-8.49330100	0.50672400	0.00089500
H	4.88316700	-1.04657000	-2.15868800
H	4.80114500	-2.57807900	-1.27311300
H	3.40895900	-1.48967100	-1.28011300
H	4.88586800	-1.04112500	2.16167700
H	4.80169100	-2.57478300	1.28001500
H	3.41023700	-1.48533800	1.28609900
C	7.38362900	-2.09204600	0.00150000
H	5.81606200	3.11740200	0.88374800
H	5.81563900	3.11566000	-0.89043500
H	7.33082900	2.89661000	-0.00348400
C	8.49330000	0.50672400	-0.00159400
C	-8.76758600	-1.90766200	-0.00176500
H	-6.95486100	-3.08749000	-0.00325800
C	-9.30780500	-0.62491400	-0.00029900
H	-8.92369600	1.49865300	0.00204100
C	8.76758600	-1.90766200	0.00080800
H	6.95486200	-3.08749000	0.00270200
C	9.30780500	-0.62491400	-0.00071200
H	8.92369500	1.49865300	-0.00279000
H	-9.42570600	-2.76626300	-0.00265500
H	-10.38242400	-0.49830000	-0.00007000
H	9.42570700	-2.76626400	0.00145700
H	10.38242400	-0.49830000	-0.00123200

Cy7 HOMO and LUMO (from g16 *cubegen*, isoval = 0.04)

Cy7 MO=110 (HOMO):

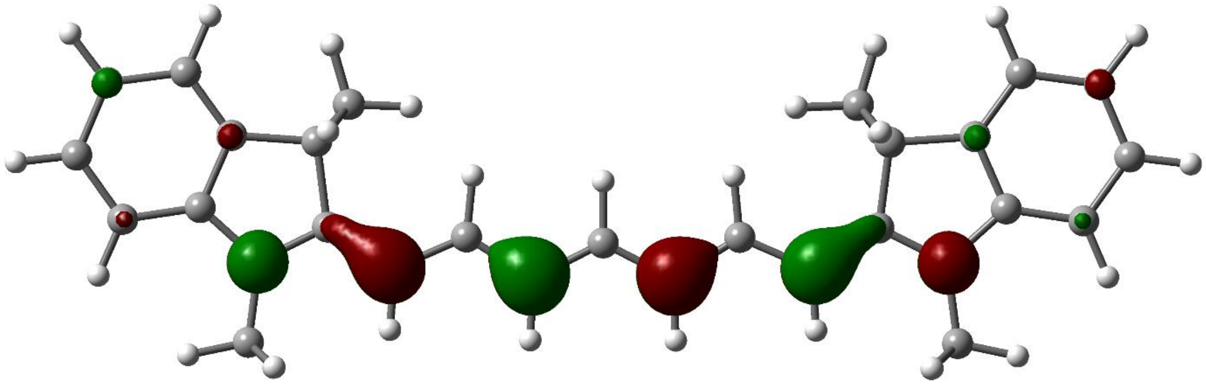

Cy7 MO=111 (LUMO):

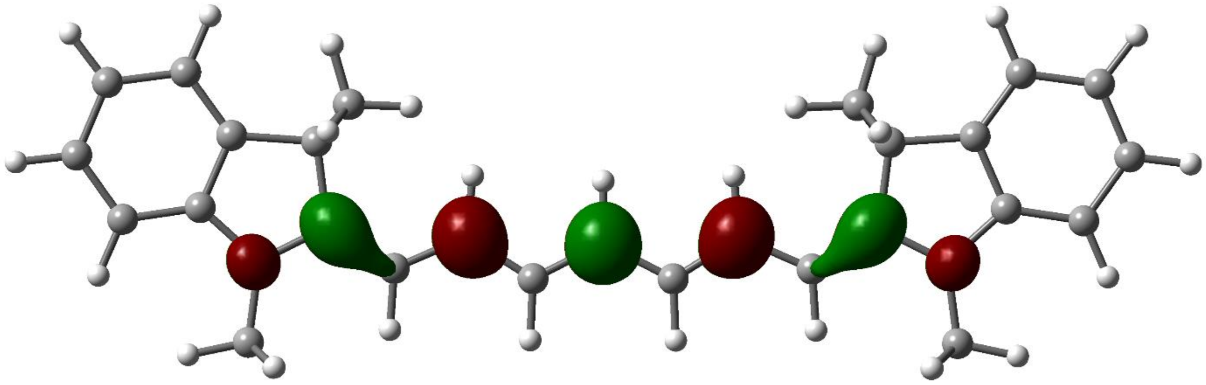

Cy7-PPG (S_0) optimized geometry (# opt=calcfc freq scrf=(smd,solvent=water) def2svp mn15)

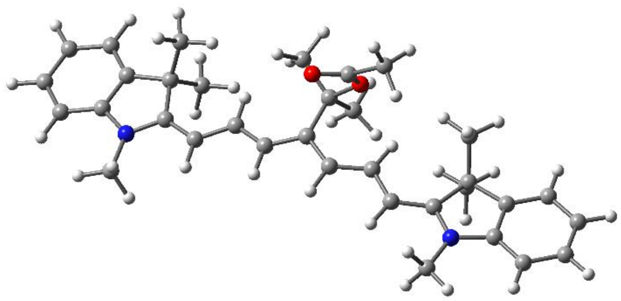

EE + Thermal Free Energy Correction: -1576.232828 Ha

1 1

C	-3.72495900	1.22963200	-0.32016400
C	-2.63158700	0.37133800	-0.17384600
C	-1.31684700	0.75164000	-0.45061200
C	-0.12416400	0.01894000	-0.32125600
C	1.07002800	0.75754000	-0.53329400
C	2.38033800	0.38781500	-0.25192300
C	3.48869900	1.22614100	-0.46735700
C	4.79126200	0.90908400	-0.09572000
C	5.29623100	-0.32261900	0.65602700
C	-5.04594600	0.86213800	-0.05079600
C	-5.57935300	-0.48509000	0.43171400
H	-2.82410700	-0.62951100	0.19742300
H	2.56513200	-0.59280300	0.18180900
N	5.83448900	1.73880900	-0.34426100
N	-6.07465300	1.72160900	-0.20922700
C	-5.92769200	3.09131800	-0.66245000
H	-5.49908400	3.11826100	-1.67430200
H	-6.90812700	3.57739200	-0.68102500
H	-5.26883700	3.65096000	0.01615400
C	5.70714600	3.01634700	-1.01459100
H	6.68650100	3.50204200	-1.06956500
H	5.32031800	2.88205400	-2.03510600
C	7.04341100	1.20172100	0.13413800

C	8.33171000	1.72698100	0.06449900
C	6.77875700	-0.03155300	0.73585900
C	9.36499100	0.96908900	0.62943100
H	8.54048300	2.68679900	-0.41037100
C	7.80924900	-0.77568400	1.29359600
C	9.11420100	-0.26507200	1.23762500
H	10.38628500	1.35391400	0.59036400
H	7.60527500	-1.74037400	1.76655500
H	9.93948300	-0.83401100	1.67015100
C	-7.30524900	1.11499200	0.11197800
C	-7.06358800	-0.20426300	0.50143200
C	-8.58865700	1.65399600	0.07839800
C	-8.11639200	-1.02911900	0.87357100
C	-9.64435000	0.81520400	0.45522000
H	-8.77478400	2.68458600	-0.22699000
C	-9.41773100	-0.50818800	0.84809400
H	-7.93214400	-2.06270200	1.17912800
H	-10.66367400	1.20659400	0.44000600
H	-10.26087000	-1.13900900	1.13624200
C	4.70310900	-0.38394700	2.07298000
H	4.87449100	0.55945400	2.61209600
H	3.62134700	-0.58404100	2.04889700
H	5.19100500	-1.19702200	2.63177400
C	5.05731800	-1.63907900	-0.09711400
H	3.99901100	-1.93466600	-0.08631800
H	5.39679600	-1.56782100	-1.14118300
H	5.62978800	-2.43771800	0.39862600
C	-5.30765800	-1.60988600	-0.57972500
H	-5.65498900	-1.32797000	-1.58448300
H	-4.23927400	-1.86502300	-0.63392900
H	-5.85559100	-2.51042500	-0.26372900
C	-5.05295000	-0.85283000	1.82778700
H	-3.98183200	-1.10230400	1.80997200
H	-5.21222600	-0.02954800	2.53936000
H	-5.59962100	-1.73606100	2.19116000
H	5.02052800	3.67414700	-0.46245500
H	3.30922700	2.19217400	-0.94695600
H	-3.53463900	2.24536900	-0.67651700
C	-0.05404600	-1.46027300	0.09489100

O	1.07184700	-2.07773900	-0.58659800
C	1.11047200	-2.04995000	-1.92501600
C	2.39503400	-2.58615300	-2.46751300
H	2.25931400	-2.89196300	-3.51079700
H	3.13417600	-1.76904400	-2.43026800
H	2.77179100	-3.41497400	-1.85573800
O	0.20919000	-1.59140800	-2.59960800
C	-1.27647700	-2.34045000	-0.18677500
H	-1.77399300	-2.09735400	-1.13344600
H	-0.91653800	-3.37851700	-0.23156700
H	-2.00203800	-2.29081300	0.63716200
C	0.28263400	-1.54716800	1.58218800
H	1.23168100	-1.04497000	1.81614600
H	-0.52097400	-1.06755900	2.15867500
H	0.35137600	-2.60337800	1.88093600
H	-1.18328200	1.79228000	-0.76740000
H	0.92380500	1.77826100	-0.90405800

Cy7-PPG (S_0TS) optimized geometry (# opt=calcfc,ts,noeigentest freq scrf=(smd,solvent=water) def2svp mn15)

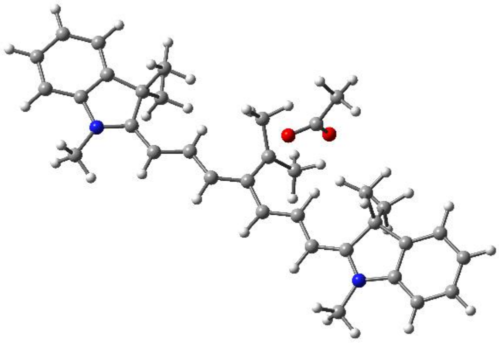

EE + Thermal Free Energy Correction: -1576.166810 Ha

1 1

C	3.82766400	-1.37594700	-0.19712800
C	2.72760100	-0.66607900	0.23135700
C	1.41463700	-1.16782700	0.07107000
C	0.18429900	-0.59621600	0.42688600
C	-1.01088800	-1.36351900	0.24813700
C	-2.28905000	-0.84864900	0.28261900
C	-3.45319100	-1.63171900	0.10080400
C	-4.74295800	-1.12834300	0.06607900
C	-5.18758900	0.33180700	0.10529300
C	5.15892200	-0.88999800	-0.11723400
C	5.61369800	0.48828500	0.35367200
H	2.88085000	0.29778000	0.70656700
H	-2.39063700	0.23081100	0.42840200
N	-5.83873400	-1.92605200	-0.05608800
N	6.21750700	-1.60765600	-0.49636500
C	6.25530400	-2.97011400	-1.00056400
H	5.27205300	-3.44167900	-0.93660700
H	6.59392300	-2.96390900	-2.04553900
H	6.96874200	-3.54974700	-0.39999500
C	-5.77546000	-3.36917900	-0.14100900
H	-6.78874200	-3.78217900	-0.17987700
H	-5.23192900	-3.68333600	-1.04441600
C	-7.02144600	-1.16846500	-0.07647700
C	-8.34310400	-1.59586600	-0.18465800
C	-6.69165300	0.18628300	0.03652300

C	-9.34084500	-0.61273500	-0.17221200
H	-8.60276900	-2.65155300	-0.27602000
C	-7.68690200	1.15324600	0.04739300
C	-9.02483900	0.74456700	-0.05755400
H	-10.38614200	-0.91852400	-0.25410600
H	-7.43065700	2.21276600	0.13530800
H	-9.82346400	1.48896200	-0.05034300
C	7.41332300	-0.86716000	-0.36575200
C	7.10431000	0.39354200	0.13917800
C	8.71021600	-1.26602600	-0.67609100
C	8.11697200	1.31841100	0.36052600
C	9.72234700	-0.32778800	-0.45230000
H	8.93034800	-2.25714600	-1.07529300
C	9.43280800	0.94442900	0.05855400
H	7.89213500	2.31142200	0.75803100
H	10.75619100	-0.59349100	-0.68109200
H	10.24578600	1.65453400	0.22175400
C	-4.78256500	1.06130500	1.39419100
H	-5.03991300	0.46557700	2.28229600
H	-3.71130600	1.30192400	1.41722900
H	-5.33136600	2.01407700	1.44797200
C	-4.68467200	1.06371800	-1.15177300
H	-3.59467600	0.97988200	-1.26057000
H	-5.14583600	0.63232400	-2.05303800
H	-4.96093800	2.12833600	-1.09498900
C	5.02776400	1.61149200	-0.51810700
H	5.22150600	1.42361600	-1.58406700
H	3.94428800	1.72513800	-0.36908200
H	5.51174500	2.55890500	-0.23818800
C	5.32352300	0.72173600	1.84705500
H	4.25246500	0.87190800	2.04276800
H	5.68397500	-0.11937700	2.45657400
H	5.85351900	1.63232500	2.16335300
H	-5.26105500	-3.78442700	0.73794400
H	-3.31776300	-2.71046500	-0.01673700
H	3.65986900	-2.35935100	-0.64082500
C	0.18796600	0.65703800	1.17535000
O	-1.82158400	2.45528800	0.13151600
C	-1.57329900	2.47469700	-1.10733900

C	-2.01509200	3.70622300	-1.88607800
H	-1.39228000	3.85533300	-2.77790400
H	-3.05295700	3.54362700	-2.22050200
H	-2.00365300	4.60373000	-1.25367100
O	-1.03429800	1.53589000	-1.74848700
C	1.03744800	1.79682300	0.81026800
H	1.44149600	1.74691900	-0.20583000
H	0.44003300	2.71083200	0.95497200
H	1.84697100	1.87441100	1.56349400
C	-0.59659100	0.76272900	2.41288100
H	-0.96196100	-0.20029400	2.78454100
H	-0.02173600	1.31863800	3.16995000
H	-1.45580100	1.41470200	2.16300500
H	1.34416100	-2.18669400	-0.32654000
H	-0.87078700	-2.41400600	-0.02734700

Cy7-PPG (S_1) optimized geometry (# opt=calcfc freq td=(root=1) scrf=(smd,solvent=water) def2svp mn15)

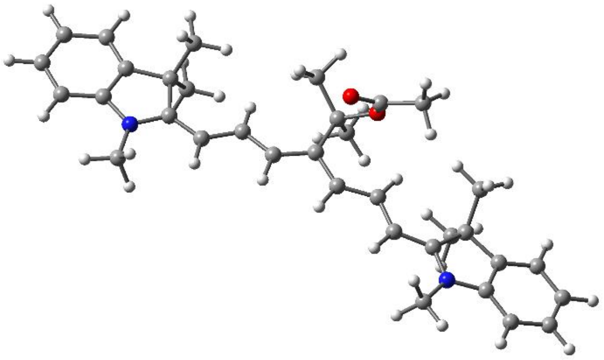

EE + Thermal Free Energy Correction: -1576.176870 Ha

1 1

C	3.70744500	1.11547600	0.27782900
C	2.64329400	0.22159500	0.06185600
C	1.32521500	0.55678600	0.39159900
C	0.13243200	-0.19188000	0.27557400
C	-1.05967200	0.55193400	0.46452200
C	-2.38602900	0.20548500	0.17377900
C	-3.45124900	1.08350600	0.44220000
C	-4.78910600	0.86877200	0.10393800
C	-5.39873200	-0.28172300	-0.68616000
C	5.05349300	0.84497000	0.02805400
C	5.68050800	-0.44171300	-0.49212300
H	2.85606600	-0.74354300	-0.38930100
H	-2.59595700	-0.75568800	-0.29218800
N	-5.76755700	1.75737400	0.43540600
N	6.03351100	1.76471800	0.25724700
C	5.78942100	3.10361500	0.75116700
H	5.35460700	3.07075100	1.76131700
H	6.73085900	3.66040800	0.78893000
H	5.09222000	3.63408500	0.08660700
C	-5.53759300	2.97190100	1.18938500
H	-6.49170300	3.47327900	1.37938900
H	-5.06277900	2.73800600	2.15318300

C	-7.01182300	1.34557900	-0.04641100
C	-8.25807300	1.96683900	0.07118900
C	-6.85280000	0.12762300	-0.72271300
C	-9.35152100	1.32282600	-0.51415500
H	-8.38372100	2.91578900	0.59442800
C	-7.94647700	-0.50188500	-1.29952900
C	-9.20585700	0.10513300	-1.19149400
H	-10.33931200	1.78247600	-0.43966900
H	-7.82596200	-1.45191100	-1.82736600
H	-10.07967400	-0.37297600	-1.63841500
C	7.29588300	1.24186100	-0.02978800
C	7.14718800	-0.07797100	-0.47966200
C	8.55107200	1.84561800	0.08195200
C	8.25988400	-0.82778200	-0.83293200
C	9.66398300	1.07970900	-0.27582000
H	8.66866800	2.87136100	0.43410100
C	9.52850000	-0.23928900	-0.72787500
H	8.14718600	-1.85688100	-1.18498300
H	10.65917200	1.52272600	-0.19871400
H	10.41748700	-0.81194200	-0.99932300
C	-4.83404300	-0.34478400	-2.11567400
H	-4.94456900	0.62346900	-2.62548200
H	-3.76992000	-0.62365700	-2.11380700
H	-5.38775600	-1.10507600	-2.68753600
C	-5.24781200	-1.64071200	0.01652600
H	-4.20803100	-1.99546300	-0.00393700
H	-5.58535400	-1.58699600	1.06225700
H	-5.86643100	-2.38324500	-0.51049000
C	5.42148500	-1.63519700	0.44218100
H	5.72560600	-1.40023500	1.47271100
H	4.36088400	-1.92715400	0.44580200
H	6.01098200	-2.49692600	0.09359600
C	5.23594600	-0.76117400	-1.92975600
H	4.17243500	-1.03683800	-1.97445300
H	5.40586300	0.09899100	-2.59371300
H	5.82467900	-1.61200500	-2.30533700
H	-4.88097100	3.65614200	0.63164300
H	-3.20471500	2.01940700	0.95331700
H	3.45712700	2.09515000	0.69722200

C	0.08196800	-1.66648900	-0.13016400
O	-1.08070000	-2.28370600	0.49466100
C	-1.20089600	-2.26648400	1.82643800
C	-2.52149500	-2.79901000	2.28432700
H	-2.88744900	-3.58822200	1.61651600
H	-2.43827200	-3.15981300	3.31572800
H	-3.24241600	-1.96558400	2.26047400
O	-0.34004000	-1.82861800	2.56529800
C	1.30016400	-2.52024900	0.24204800
H	1.76715000	-2.21102600	1.18537800
H	0.95364100	-3.55934500	0.34130100
H	2.05226700	-2.50928200	-0.55847600
C	-0.18326700	-1.77877700	-1.62859700
H	-1.11449000	-1.26431800	-1.90763600
H	0.65160700	-1.31513300	-2.17379400
H	-0.25487500	-2.83705000	-1.92082200
H	1.18372400	1.58068600	0.75946600
H	-0.91224200	1.56687100	0.85357800

Cy7-PPG (S_1TS) optimized geometry (# opt=(calcfc,ts,noeigentest) freq td=(root=1) scrf=(smd,solvent=water) def2svp mn15)

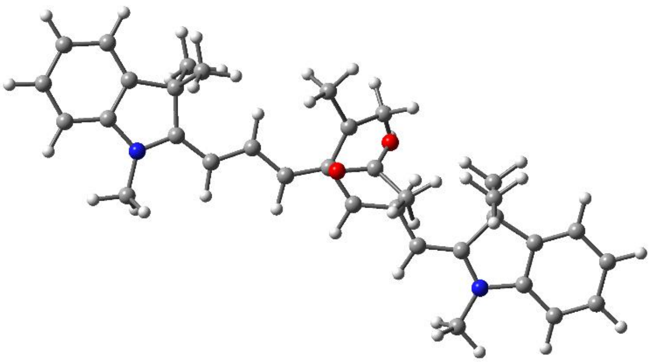

EE + Thermal Free Energy Correction: -1576.142776 Ha

1 1

C	3.72427500	1.13547400	-0.10078500
C	2.65517100	0.26857900	-0.35690400
C	1.33075200	0.70222900	-0.29832200
C	0.11980700	-0.02102300	-0.51341800
C	-1.05525800	0.79619500	-0.50430700
C	-2.37701100	0.36204500	-0.42670300
C	-3.45976400	1.24780200	-0.36570400
C	-4.80196700	0.84833600	-0.29741800
C	-5.37386000	-0.56346900	-0.31005100
C	5.07404800	0.76074200	-0.12496700
C	5.67292200	-0.61074100	-0.40350000
H	2.87540000	-0.75946400	-0.62883000
H	-2.56460200	-0.70748600	-0.37617600
N	-5.81060100	1.74501600	-0.23213700
N	6.06430000	1.64438100	0.13174200
C	5.84636800	3.04364300	0.44642000
H	5.24173400	3.13888600	1.35936800
H	6.80913000	3.53782200	0.60700100
H	5.32226100	3.54292500	-0.38056700
C	-5.62519300	3.18408400	-0.21638700
H	-6.60088200	3.67818600	-0.18695500
H	-5.04469900	3.48458500	0.66716000

C	-7.05892000	1.10674300	-0.19015900
C	-8.33462200	1.66746600	-0.11323500
C	-6.85434800	-0.27701300	-0.23692600
C	-9.41694100	0.78415500	-0.08783500
H	-8.49198100	2.74602900	-0.07191200
C	-7.93704900	-1.14526800	-0.21107800
C	-9.22716300	-0.60327900	-0.13681700
H	-10.42985400	1.18731900	-0.02787100
H	-7.78375700	-2.22707700	-0.24774100
H	-10.09362200	-1.26709100	-0.11580500
C	7.32403000	1.03188300	0.06610200
C	7.14663600	-0.32101500	-0.24711800
C	8.58854000	1.58901000	0.26277200
C	8.24487800	-1.16077800	-0.37052600
C	9.68677100	0.73424600	0.13624600
H	8.72621500	2.64360100	0.50497400
C	9.52373500	-0.62206600	-0.17512100
H	8.11263000	-2.21817500	-0.61471000
H	10.69136400	1.13580000	0.28372700
H	10.40211400	-1.26390700	-0.26620800
C	-5.04346600	-1.30156600	-1.61871500
H	-5.32650600	-0.69972600	-2.49459200
H	-3.97299100	-1.54531300	-1.68511200
H	-5.61065100	-2.24419100	-1.64568400
C	-4.93877300	-1.38999700	0.91192300
H	-3.87125000	-1.64856100	0.87368600
H	-5.13889700	-0.84735400	1.84763900
H	-5.51512000	-2.32754100	0.92369500
C	5.22531600	-1.65637700	0.63173100
H	5.41112000	-1.30198600	1.65602700
H	4.15729300	-1.89840600	0.52928600
H	5.80084700	-2.58074500	0.47270700
C	5.38484400	-1.09121500	-1.83590000
H	4.32378700	-1.34282100	-1.97531700
H	5.66893500	-0.32633200	-2.57305200
H	5.97639200	-1.99852800	-2.03000700
H	-5.09068200	3.50771400	-1.12049900
H	-3.24195500	2.31955500	-0.37873700
H	3.48583400	2.17337300	0.14915000

C	0.03057100	-1.45527200	-0.65695200
O	-1.15205600	-1.88438100	1.14814700
C	-0.90168600	-1.14369100	2.16160200
C	-2.08663000	-0.81518000	3.05320700
H	-2.73348100	-1.69262300	3.19100900
H	-1.74815000	-0.43348000	4.02473900
H	-2.68758400	-0.03522200	2.55650400
O	0.21555500	-0.66307200	2.43327400
C	1.13854700	-2.37364700	-0.26556400
H	1.66528000	-2.03150000	0.63404800
H	0.72314000	-3.37603200	-0.09285400
H	1.85944300	-2.46642000	-1.09860200
C	-0.94823400	-2.06238600	-1.61410900
H	-1.49832300	-1.32045800	-2.20576800
H	-0.38992500	-2.71947000	-2.30057400
H	-1.66678300	-2.70353200	-1.07868200
H	1.19559300	1.77077300	-0.09396300
H	-0.88864800	1.87880100	-0.48802900

Cy7-PPG (T_1) optimized geometry (# opt=calcfc freq scrf=(smd,solvent=water) def2svp mn15)

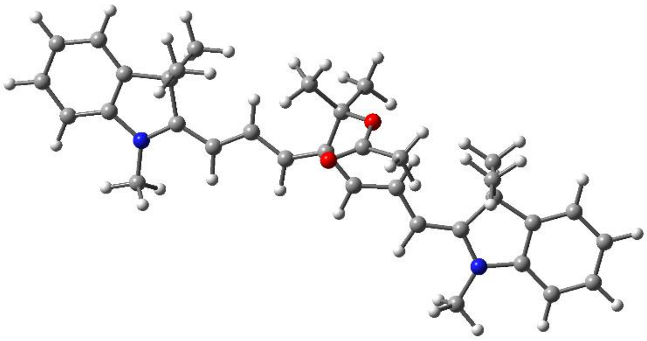

EE + Thermal Free Energy Correction: -1576.196286 Ha

1 3

C	3.67940900	1.05614800	0.21232900
C	2.65116700	0.12710000	-0.01960400
C	1.31684500	0.45586400	0.25773100
C	0.12633200	-0.28872100	0.14190900
C	-1.05515500	0.47051800	0.33331500
C	-2.39565500	0.13269100	0.10703700
C	-3.41896700	1.06437100	0.35683700
C	-4.77560500	0.88289100	0.08346200
C	-5.45495500	-0.29588800	-0.60081300
C	5.04188000	0.83092100	0.01508300
C	5.72905900	-0.43935500	-0.46951500
H	2.90143100	-0.84694300	-0.42789900
H	-2.64789200	-0.84812100	-0.29124700
N	-5.70440100	1.83137200	0.38657700
N	5.97928100	1.78930700	0.26477600
C	5.67251500	3.12629200	0.72947700
H	5.20917800	3.09465900	1.72632500
H	6.59272800	3.71593900	0.78700400
H	4.98102700	3.62337800	0.03464900
C	-5.40229100	3.08937800	1.03878300
H	-6.33176300	3.63056300	1.24116800
H	-4.88594800	2.90954400	1.99196700

C	-6.98496200	1.43071100	-0.02008100
C	-8.19940900	2.10755100	0.09592300
C	-6.89494700	0.16343900	-0.60779600
C	-9.33919800	1.46564100	-0.39824200
H	-8.26891000	3.09714800	0.54935500
C	-8.03309100	-0.46470600	-1.09485000
C	-9.26403800	0.19679500	-0.98577700
H	-10.30561900	1.96827900	-0.32243800
H	-7.96770500	-1.45470100	-1.55436300
H	-10.17148300	-0.27908400	-1.36244900
C	7.27189700	1.30331400	0.02465800
C	7.18163900	-0.02319400	-0.41418800
C	8.49939000	1.95022800	0.17099000
C	8.33046100	-0.73815000	-0.72409900
C	9.65033300	1.22005900	-0.14297200
H	8.57004900	2.98266300	0.51637200
C	9.57414600	-0.10594100	-0.58532500
H	8.26407300	-1.77384400	-1.06842400
H	10.62643800	1.69835700	-0.03825100
H	10.49005100	-0.65057900	-0.82273900
C	-4.95316200	-0.46979000	-2.04392900
H	-5.05550200	0.46700600	-2.61118500
H	-3.89887300	-0.78319200	-2.06653100
H	-5.55287800	-1.24631500	-2.54275800
C	-5.31623900	-1.60809100	0.18559700
H	-4.29159200	-2.00171800	0.13938000
H	-5.59828500	-1.47184100	1.24033800
H	-5.98477600	-2.36173500	-0.25783700
C	5.48269600	-1.63102300	0.46904000
H	5.74228200	-1.37406200	1.50655900
H	4.43448800	-1.96388700	0.44066600
H	6.11485900	-2.47448000	0.15198700
C	5.33973900	-0.78578000	-1.91576200
H	4.28533800	-1.08849000	-1.99018100
H	5.50806000	0.07173500	-2.58371700
H	5.96096800	-1.62489500	-2.26442900
H	-4.75963900	3.71252700	0.40021400
H	-3.11911600	2.02130600	0.79501900
H	3.38218400	2.03605000	0.59905000

C	0.04805500	-1.76210800	-0.25892000
O	-1.08088000	-2.37434300	0.43397800
C	-1.14153500	-2.34848900	1.76884200
C	-2.44505800	-2.87056900	2.28624900
H	-2.31651000	-3.23975900	3.31006700
H	-3.15819900	-2.03058200	2.30341400
H	-2.85020100	-3.65150900	1.63152100
O	-0.24745600	-1.91436600	2.46979800
C	1.27945800	-2.62128800	0.03985600
H	1.76507700	-2.35591600	0.98711900
H	0.94359900	-3.66727700	0.09379600
H	2.01107800	-2.56013200	-0.77724300
C	-0.30686300	-1.88210900	-1.73781000
H	-1.24600000	-1.35727200	-1.96649200
H	0.49967200	-1.43402200	-2.33562800
H	-0.41066500	-2.94106200	-2.01813500
H	1.16678400	1.48834100	0.59786300
H	-0.88863200	1.49657600	0.68380500

Cy7-PPG (T$_1$TS) optimized geometry (# opt=(calcfc,ts,noeigentest) freq scrf=(smd,solvent=water) def2svp mn15)

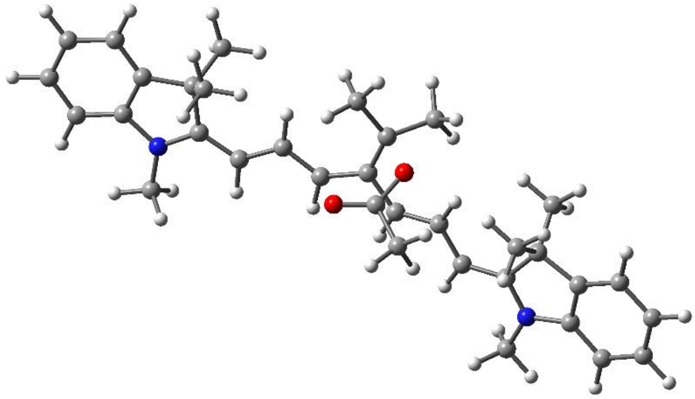

EE + Thermal Free Energy Correction: -1576.165972 Ha

1 3

C	3.68087200	1.08522500	-0.19165200
C	2.64248900	0.17146500	-0.43605400
C	1.31816400	0.59696500	-0.47170500
C	0.09397000	-0.11229700	-0.69191900
C	-1.06869300	0.72787600	-0.63009900
C	-2.39203000	0.32418400	-0.49401700
C	-3.44808700	1.24138500	-0.39074200
C	-4.79700100	0.88143700	-0.26806200
C	-5.40620300	-0.51454600	-0.25974900
C	5.04063500	0.76081700	-0.12715000
C	5.70043200	-0.59963700	-0.30559200
H	2.89510700	-0.87005800	-0.60612600
H	-2.60597400	-0.74005800	-0.43740600
N	-5.77401300	1.80641000	-0.15974900
N	5.98411600	1.69560500	0.12555300
C	5.70349200	3.09978900	0.36251900
H	5.02970600	3.21181600	1.22294300
H	6.63788300	3.62757400	0.57526400
H	5.23206600	3.55007700	-0.52189500
C	-5.55019900	3.24110600	-0.16102200
H	-6.51169600	3.76107000	-0.11613200
H	-4.94340300	3.53340900	0.70695600

C	-7.03889400	1.19929200	-0.05130600
C	-8.29115100	1.79493400	0.09258500
C	-6.87261600	-0.18751200	-0.10638200
C	-9.39477300	0.94122800	0.17636900
H	-8.41548400	2.87743700	0.14079700
C	-7.97569900	-1.02689500	-0.02304700
C	-9.24455800	-0.45025500	0.11865200
H	-10.39175900	1.37156000	0.28966200
H	-7.85385700	-2.11238600	-0.06647400
H	-10.12607900	-1.09068100	0.18601500
C	7.27080300	1.12774900	0.13806000
C	7.15548000	-0.24290500	-0.11314300
C	8.50311700	1.74374800	0.35294200
C	8.28883200	-1.04413500	-0.15371100
C	9.63773900	0.92814800	0.31006700
H	8.59168300	2.81352500	0.54688100
C	9.53770800	-0.44651100	0.06127300
H	8.20585000	-2.11650200	-0.34934400
H	10.61977500	1.37626300	0.47409600
H	10.44242100	-1.05687800	0.03447700
C	-5.16515600	-1.24104400	-1.59390000
H	-5.48713200	-0.62276900	-2.44459400
H	-4.10426300	-1.50117600	-1.72427000
H	-5.74941500	-2.17351000	-1.60003800
C	-4.92945600	-1.37131000	0.92396300
H	-3.88135500	-1.68130800	0.80987500
H	-5.03952700	-0.83106300	1.87587000
H	-5.54655600	-2.28147000	0.96781700
C	5.25612800	-1.59947000	0.77440400
H	5.39345600	-1.17860600	1.78108000
H	4.20198000	-1.88789100	0.65237200
H	5.87002700	-2.50884900	0.69041500
C	5.48131100	-1.16949800	-1.71595100
H	4.43320900	-1.45827300	-1.87951800
H	5.77255800	-0.43982000	-2.48545000
H	6.10388300	-2.06882500	-1.83594800
H	-5.02865000	3.54096700	-1.07997900
H	-3.20058900	2.30624100	-0.41183200
H	3.40129700	2.13039500	-0.03143700

C	-0.03485300	-1.53544200	-0.85383700
O	-1.08486100	-2.05796900	0.85959000
C	-0.77677100	-1.41754700	1.92989400
C	-1.91303500	-1.19049700	2.90950200
H	-1.52146800	-0.93671600	3.90259500
H	-2.51946800	-0.34542400	2.54381800
H	-2.56927800	-2.06950600	2.96853100
O	0.35056000	-0.95906600	2.18379600
C	1.10863800	-2.47148900	-0.61965600
H	1.65148800	-2.23748400	0.30393600
H	0.72508000	-3.49944000	-0.56638900
H	1.80612400	-2.42475100	-1.47471100
C	-1.04749200	-2.08780500	-1.81942800
H	-1.67093800	-1.31986600	-2.29231600
H	-0.49218600	-2.61425800	-2.61300500
H	-1.69159300	-2.83398800	-1.33089500
H	1.18547500	1.67642300	-0.33016400
H	-0.88356400	1.80766300	-0.61583600

2. Synthetic Methods

2.1 General remarks

All reactions were performed without excluding moisture or air, unless otherwise stated. Magnetic stirring was used for all reactions. Standard Schlenk techniques employing nitrogen as the inert gas were used for reactions requiring an inert atmosphere. Reagents were purchased from commercial suppliers and were used without further purification. Technical and analytical grade solvents were purchased from Boom B.V. or Sigma-Aldrich. Anhydrous acetonitrile was produced by drying reagent-grade acetonitrile for 24 h over activated 3 Å molecular sieves (10% w/v). Flash chromatography was performed on silica gel (Supelco, silica gel 60) with a particle size of 40−64 μM employing technical grade solvents.TLC analysis was conducted on TLC aluminum foils with a silica gel matrix (Supelco, silica gel 60) with detection by UV (254 nm or 366 nm) or by staying with PMA (phosphomolybdic acid) stain.

Nuclear magnetic resonance (NMR) spectra were recorded on an Agilent Technologies 400-MR (400/54 Premium Shielded) spectrometer (400 MHz for ^{1}H nucleus, 101 MHz for ^{13}C nucleus). Deuterated solvents *d6*-DMSO, *d3*-MeOD and $CDCl_3$ were purchased from Sigma-Aldrich. In each NMR spectrum, the chemical shift of compound resonances are given in parts per million (ppm) (δ) relative to the residual solvent proton or carbon resonance. All spectra were measured at ambient temperature. ^{1}H NMR data are reported as: chemical shift, multiplicity (s = singlet, d = doublet, t = triplet, q = quartet, m = multiplet, dd = doublet of doublets, dq = doublet of quartets, br = broad), coupling constants (J) given in Hz, and integration. ^{13}C NMR or ^{13}C-APT spectra were conducted with proton decoupling and are reported as: chemical shift, type of carbon (quat. C, CH, CH_2, CH_3).

High resolution mass spectra (HRMS) were recorded on a Thermofisher LTQ Orbitrap XL with eluent MeOH (0.1% TFA) and flow rate of 0.15 mL min-1 in positive (ACPI/ESI) mode. Melting point ranges were determined on a Stuart analogue capillary melting point SMP11 apparatus. UV/Vis spectra were recorded on an Agilent 8453, Raw data were processed using Agilent UV-Vis ChemStation B.02.01 SP1, Spectragryph 1.2 and MS Excel.

2.2 Synthetic Methods[4]

Scheme S1. An efficient synthesis toward Cy7-PPG, with two methods for loading ZS-OH with a payload.

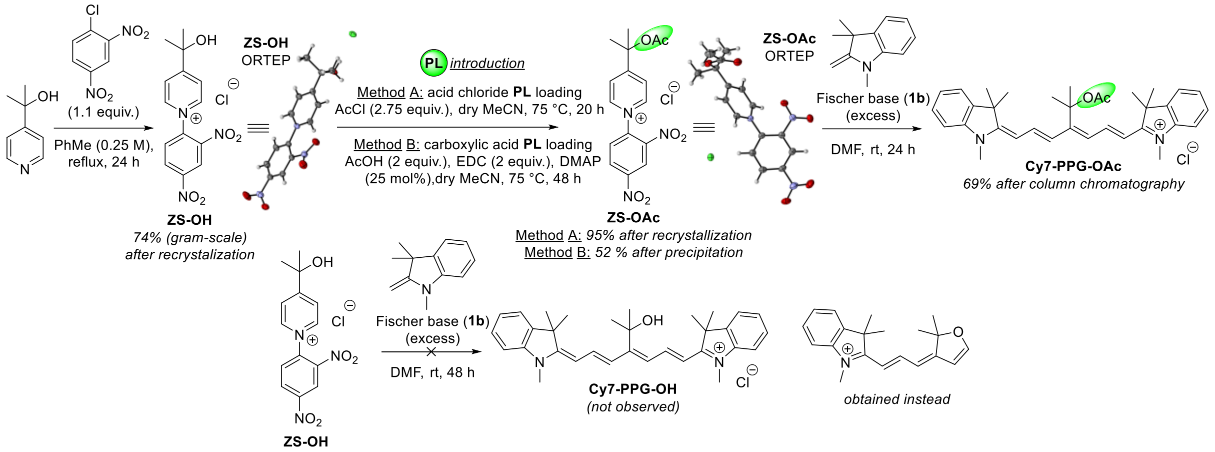

Synthesis of ZS-OH: A round-bottom flask was charged with a magnetic stirring bar, and a reflux condenser. Subsequently, 2-(4-pyridyl)-2-propanol (0.69 g, 5.0 mmol, 1.0 equiv.), 1-chloro-2,4-dinitrobenzene (1.27 g, 6.25 mmol, 1.25 equiv) and toluene (20 ml) were added to the flask and the reaction mixture was heated to reflux for 24 h. Upon cooling the reaction was deemed complete by confirming the disappearance of the limiting reactant by TLC (Rf = 0.45 in EtOAc). The solids formed upon cooling were filtered and washed with PhMe (10 ml) and Et_2O (10 ml). The filtrate was discarded, and the solids were collected, dissolved in MeOH and concentrated on the rotary evaporator. The solid residue (~1.5 g) was recrystallized from boiling MeCN (~7.5 ml) to give **ZS-OH** (1.27 g, 74 % yield) as amber crystals, which were suitable for x-ray crystallography. **^{1}H NMR** (400 MHz, CD_3OD) δ 9.27 (d, J = 2.5 Hz, 1H), 9.21 (d, J = 6.9 Hz, 2H), 8.91 (dd, J = 8.7, 2.5 Hz, 1H), 8.48 (d, J = 6.9 Hz, 2H), 8.32 (d, J = 8.7 Hz, 1H), 1.71 (s, 6H). **^{13}C NMR** (101 MHz, CD_3OD) δ 175.1, 151.1, 146.6, 144.7, 140.0, 132.7, 131.1, 125.6, 123.2, 73.0, 30.9. **MP**: 121 °C; **HRMS** (ESI): Calculated for C14H14N3O5+ ([M+]): m/z 304.0928; found: 304.0930.

Synthesis of ZS-OAc, Method A: A flame-dried round-bottom flask was charged with a magnetic stirring bar and was purged three times with nitrogen. The septum was briefly opened, and the reaction flask was charged with **ZS-OH** (170 mg, 0.50 mmol, 1.0 equiv.) and purged again with nitrogen. Dry MeCN (25 ml) was added via syringe, followed by acetyl chloride (0.100 ml, 108 mg, 2.75 equiv.). The turbid reaction mixture was heated to 75 °C under the inert atmosphere of nitrogen for 20 h. By the time of completion, the reaction mixture had turned completely clear. MeOH (25 ml) was added, and the reaction mixture was concentrated to dryness on the rotary evaporator. The reaction mixture was co-evaporated to dryness again with MeOH (25 ml), once with PhMe (25 ml) and finally once with MeCN (25 ml). The solid residue (~200 mg) was recrystallized from boiling MeCN to give **ZS-OAc** (181 mg, 95%) as amber crystals, which were suitable for x-ray crystallography. **^{1}H NMR** (400 MHz, CD_3OD) δ 9.28 (d, J = 2.5 Hz, 1H), 9.27 – 9.23 (m, 2H), 8.93 (dd, J = 8.7, 2.5 Hz, 1H), 8.43 – 8.37 (m, 2H), 8.33 (d, J = 8.7 Hz, 1H), 2.19 (s, 3H), 1.93 (s, 6H). **^{13}C NMR** (101 MHz, CD3OD) δ 171.6, 170.7, 151.2, 147.0, 139.9, 132.7, 131.1, 125.6, 125.3, 123.2, 80.8, 27.8, 21.4. **MP**: 159 °C (decomposition); **HRMS** (ESI): Calculated for C16H16N3O6+ ([M+]): m/z 346.1034; found: 346.1038.

Synthesis of ZS-OAc, Method B: A flame-dried round-bottom flask was charged with a magnetic stirring bar and was purged three times with nitrogen. The septum was briefly opened, and the reaction flask was charged with EDC (77 mg, 0.40 mmol, 2.0 equiv.) and

DMAP (6 mg, 0.05 mmol, 0.25 equiv.) and purged again with nitrogen. Dry MeCN (4 ml) was added via syringe, followed by acetic acid (23 µl, 23 mg, 2.0 equiv.). The reaction mixture was stirred at room temperature for 1h, then the septum was briefly opened and **ZS-OH** (68 mg, 0.2 mmol, 1 equiv.) was added. The turbid reaction mixture was heated to 75 °C under the inert atmosphere of nitrogen for 48 h. By the time of completion, the reaction had turned completely clear. MeOH (25 ml) was added, and the reaction mixture was concentrated to dryness on the rotary evaporator. The reaction mixture was coevaporated to dryness again with MeOH (25 ml), once with PhMe (25 ml) and finally once with MeCN (25 ml). The solid residue (~125 mg) was dissolved in boiling MeCN and upon cooling was precipitated with cold PhMe to give **ZS-OAc** (40 mg, 52%) as a dark brown powder. The analytical data matches the original sample obtained from Method A.

Synthesis of Cy7-PPG-OAc: A round-bottom flask was charged with a magnetic stirring bar. Subsequently **ZS-OAc** (76 mg, 0.20 mmol, 1 equiv.), 1,3,3-trimethyl-2-methyleneindoline (347 mg, 2.00 mmol, 10 equiv.) and DMF (1 ml) were added, and the reaction color instantly changed from a light pink to an increasingly dark green. The reaction mixture was stirred for 24 h, with LCMS showing >90% conversion at this time, based on consumption of **ZS-OAc**. Et_2O (19 ml) was added, and the turbid dark green mixture was stored at 4 °C for 2 h. The cold mixture was then filtered over a filter paper, and the dark green solids were dissolved in DCM (50 ml) and concentrated on the rotary evaporator to yield a dark green gum (~110 mg). This crude material was purified by flash column chromatography (5 g SiO_2), collecting fractions of 5 ml and eluting DCM (25 ml), 2% MeOH/DCM (25 ml) and finally 4% (MeOH/DCM) (100 ml). Fractions containing **Cy7-PPG-OAc** by TLC (Rf 0.3 in 5% MeOH/DCM, brightly green under visible light) were concentrated to yield a dark golden green film (75 mg, 69%). **^{1}H NMR** (400 MHz, CD3OD) δ 7.99 (t, *J* = 13.2 Hz, 2H), 7.45 (dd, *J* = 7.4, 1.2 Hz, 2H), 7.39 (td, *J* = 7.7, 1.2 Hz, 2H), 7.31 – 7.18 (m, 4H), 6.66 (d, *J* = 12.7 Hz, 2H), 6.45 (d, *J* = 13.5 Hz, 2H), 3.63 (s, 6H), 2.01 (s, 3H), 1.77 (s, 6H), 1.63 (s, 12H). **^{13}C NMR** (101 MHz, CD3OD) δ 173.4, 171.3, 167.6, 147.6, 144.5, 142.1, 129.7, 123.0, 123.2, 121.4, 111.7, 106.1, 83.4, 50.2, 31.8, 28.4, 28.3, 21.8. **HRMS** (ESI): Calculated for C34H41N2O2+ ([M+]): m/z 509.3163; found: 509.3154.

2.3 NMR Spectra

ZS-OH

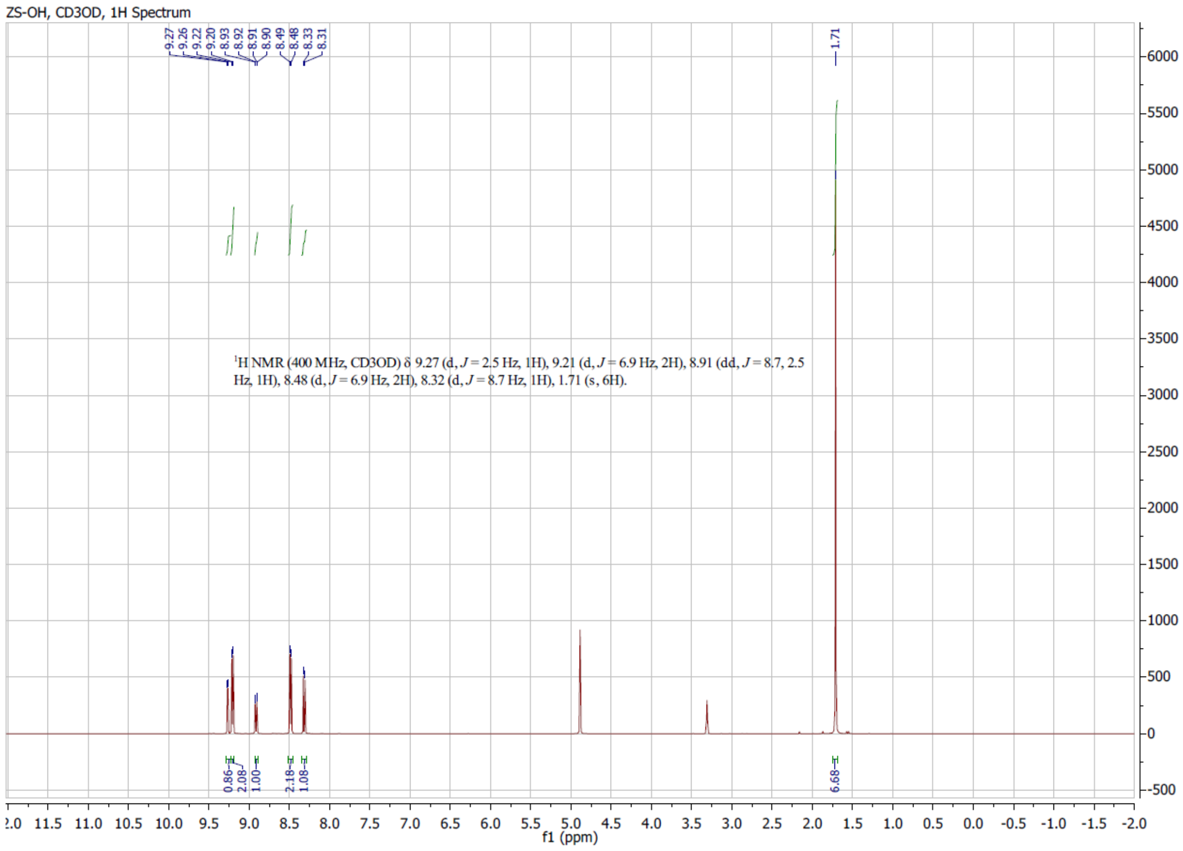

^{1}H NMR (400 MHz, CD3OD) δ 9.27 (d, *J* = 2.5 Hz, 1H), 9.21 (d, *J* = 6.9 Hz, 2H), 8.91 (dd, *J* = 8.7, 2.5 Hz, 1H), 8.48 (d, *J* = 6.9 Hz, 2H), 8.32 (d, *J* = 8.7 Hz, 1H), 1.71 (s, 6H).

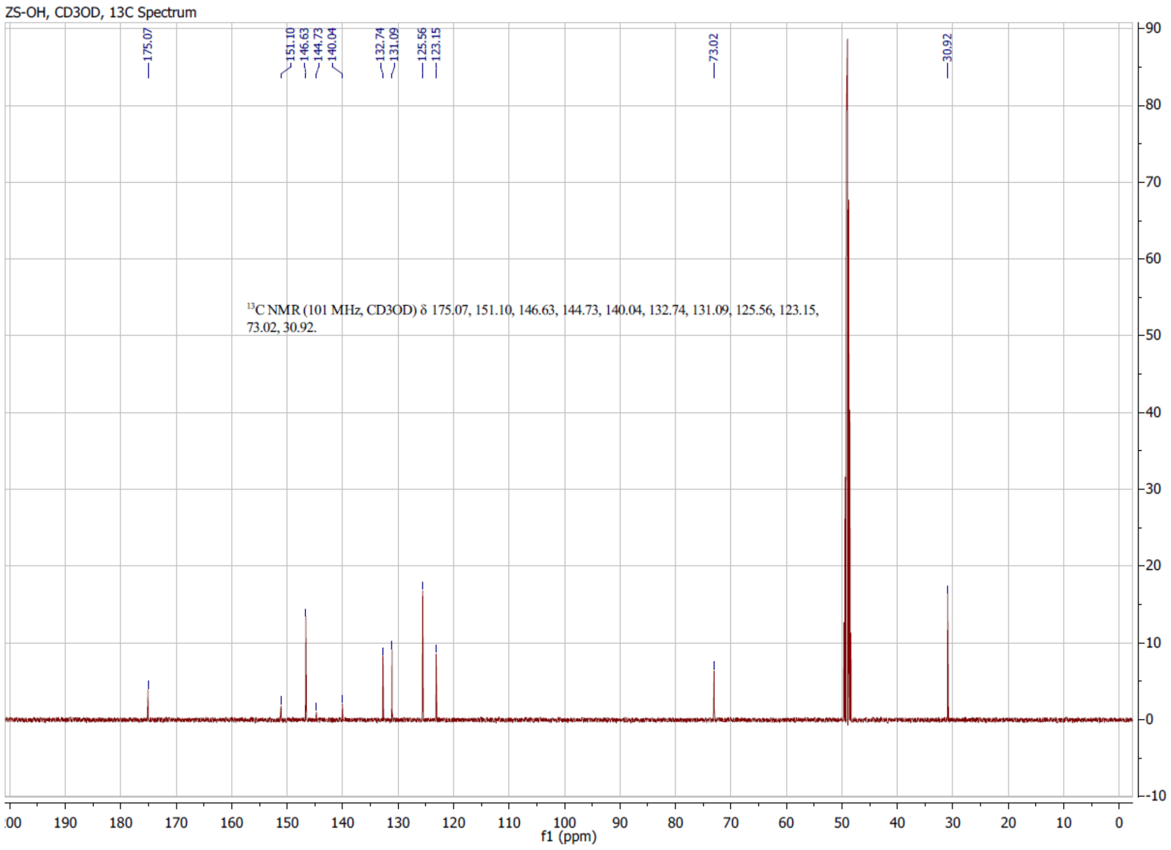

^{13}C NMR (101 MHz, CD3OD) δ 175.07, 151.10, 146.63, 144.73, 140.04, 132.74, 131.09, 125.56, 123.15, 73.02, 30.92.

ZS-OAc

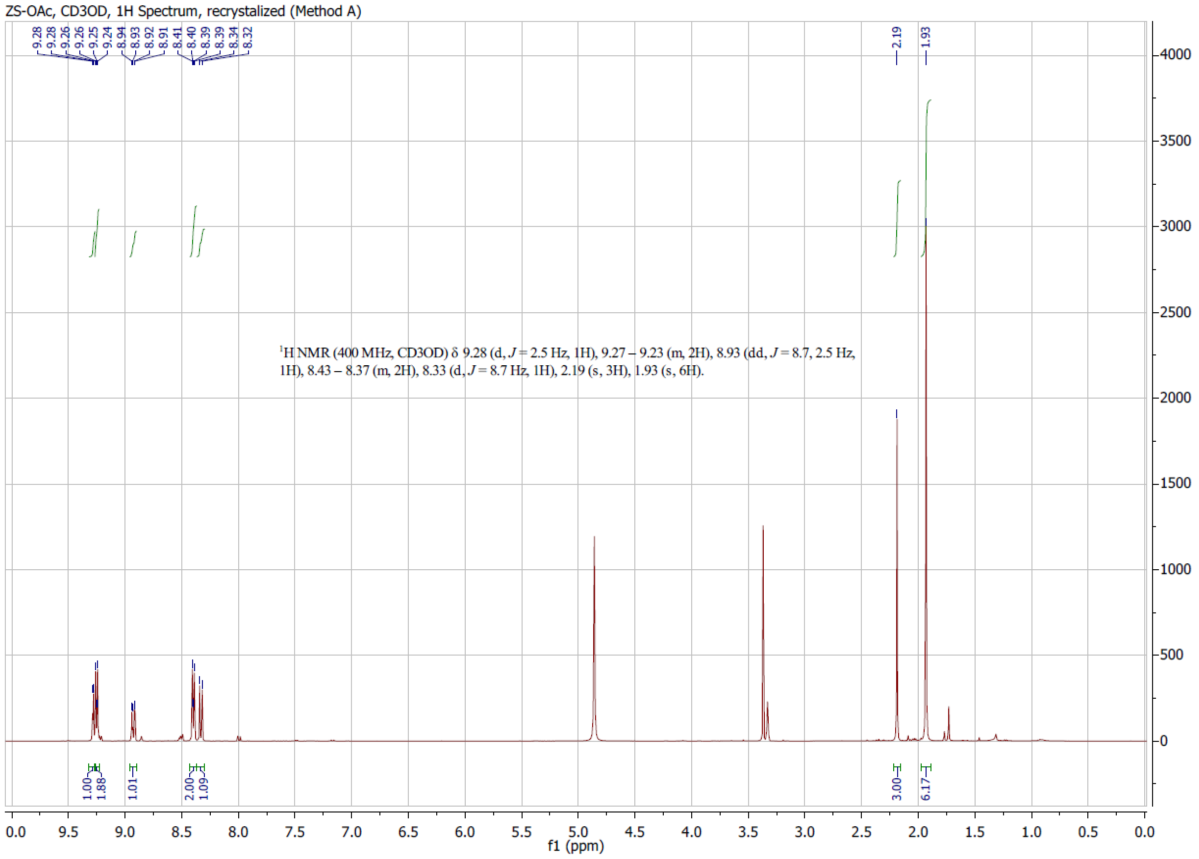

ZS-OAc, CD3OD, 1H Spectrum, recrystalized (Method A)
^{1}H NMR (400 MHz, CD3OD) δ 9.28 (d, J = 2.5 Hz, 1H), 9.27 – 9.23 (m, 2H), 8.93 (dd, J = 8.7, 2.5 Hz, 1H), 8.43 – 8.37 (m, 2H), 8.33 (d, J = 8.7 Hz, 1H), 2.19 (s, 3H), 1.93 (s, 6H).
f1 (ppm)

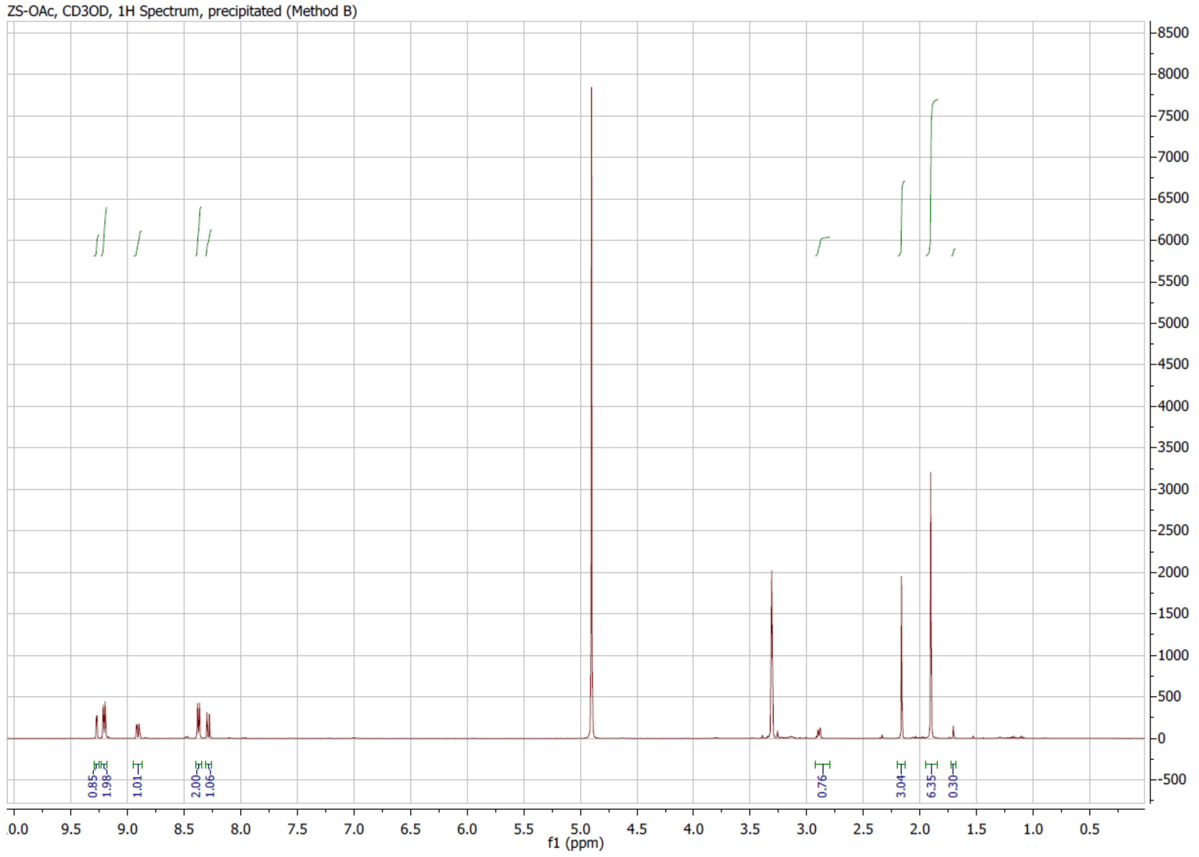

ZS-OAc, CD3OD, 1H Spectrum, precipitated (Method B)
f1 (ppm)

ZS-OAc, CD3OD, 13C Spectrum

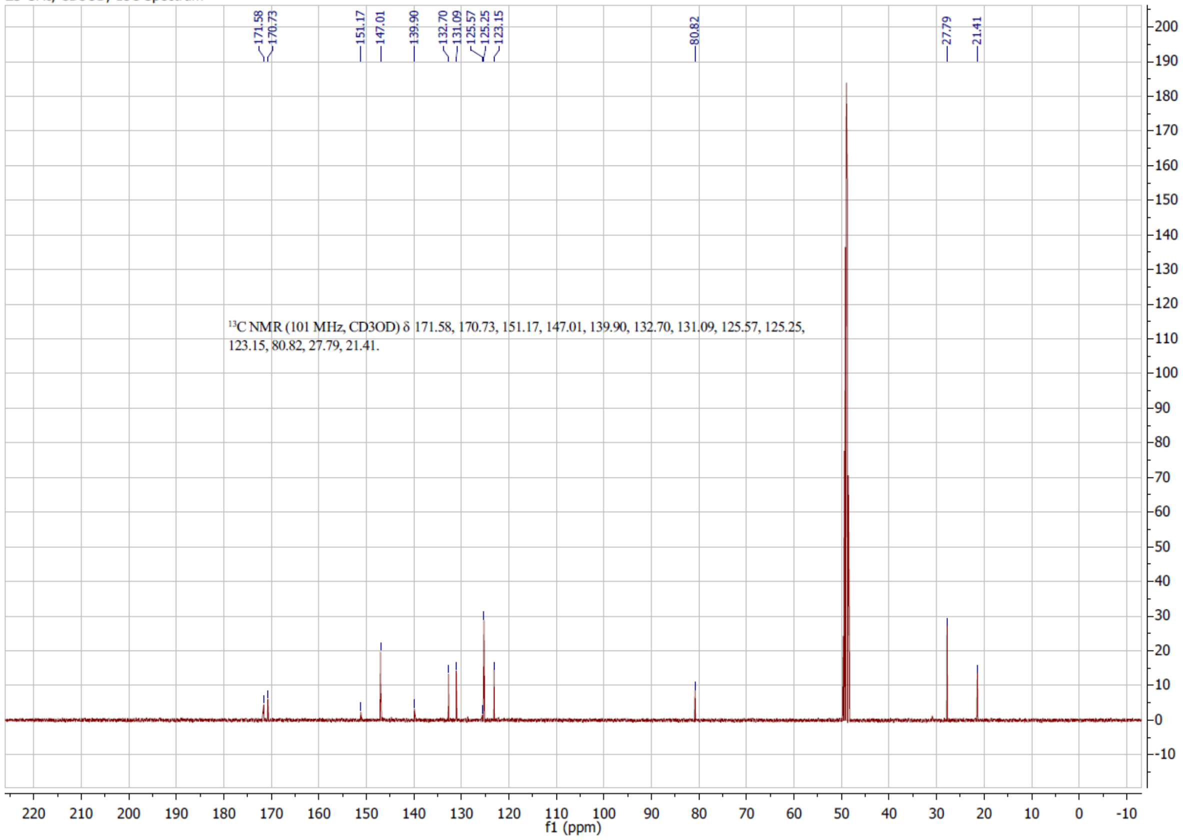

ZS-OAc, CD3OD, 13C APT Spectrum

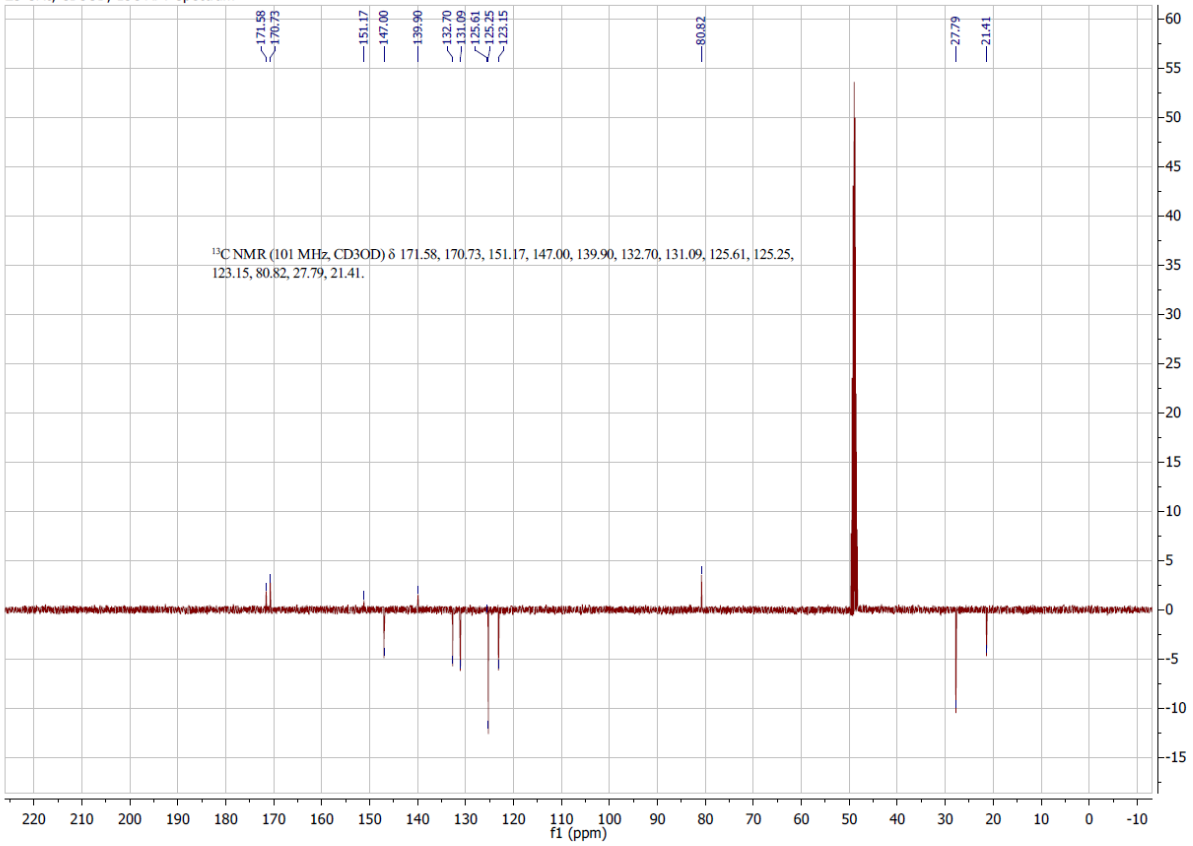

Cy7-PPG-OAc

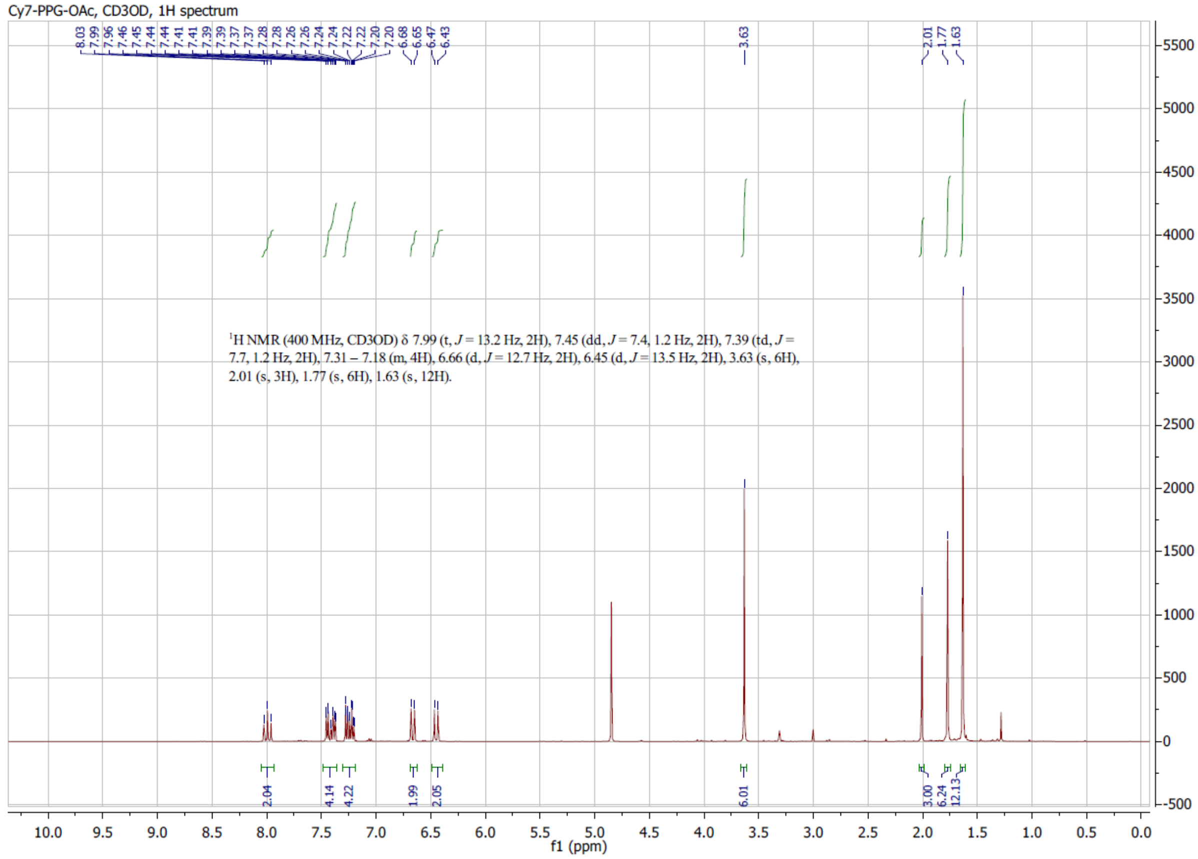
Cy7-PPG-OAc, CD3OD, 1H spectrum
^{1}H NMR (400 MHz, CD3OD) δ 7.99 (t, J = 13.2 Hz, 2H), 7.45 (dd, J = 7.4, 1.2 Hz, 2H), 7.39 (td, J = 7.7, 1.2 Hz, 2H), 7.31 – 7.18 (m, 4H), 6.66 (d, J = 12.7 Hz, 2H), 6.45 (d, J = 13.5 Hz, 2H), 3.63 (s, 6H), 2.01 (s, 3H), 1.77 (s, 6H), 1.63 (s, 12H).
f1 (ppm)

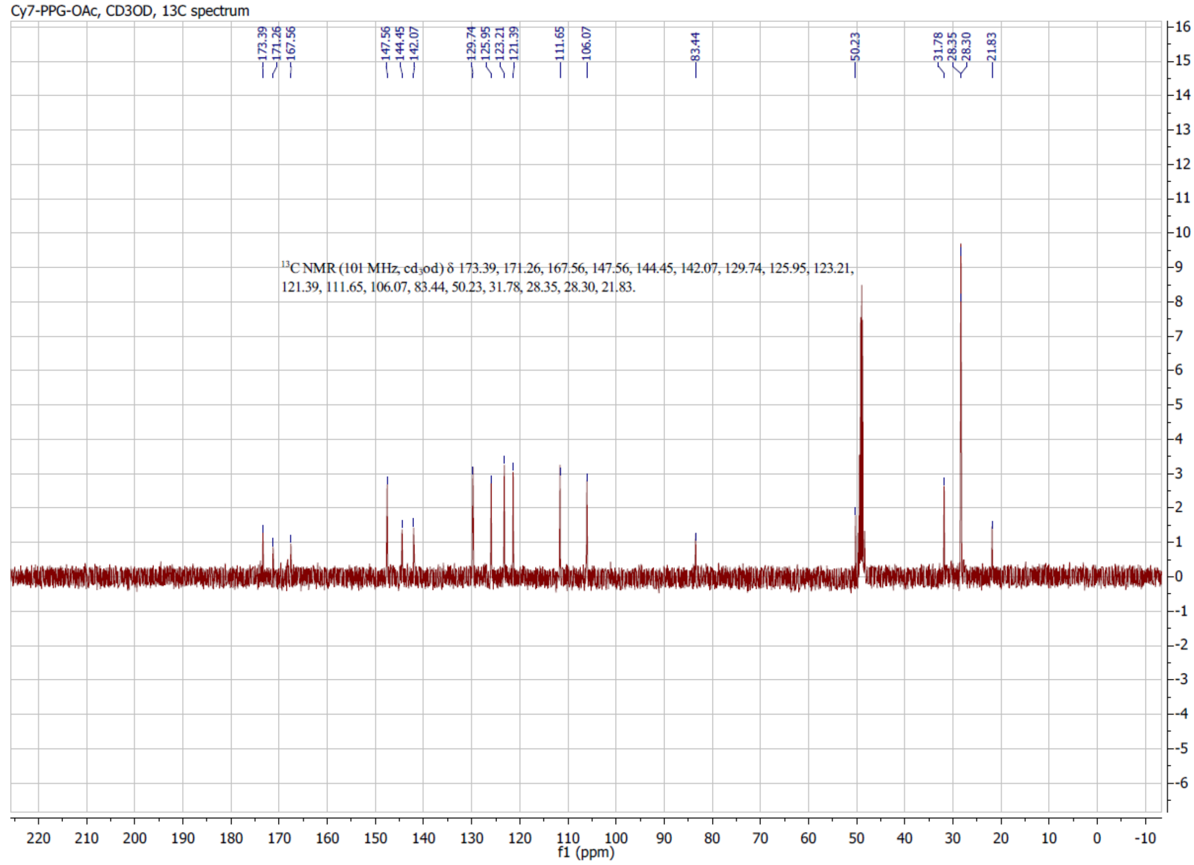
Cy7-PPG-OAc, CD3OD, 13C spectrum
^{13}C NMR (101 MHz, cd3od) δ 173.39, 171.26, 167.56, 147.56, 144.45, 142.07, 129.74, 125.95, 123.21, 121.39, 111.65, 106.07, 83.44, 50.23, 31.78, 28.35, 28.30, 21.83.
f1 (ppm)

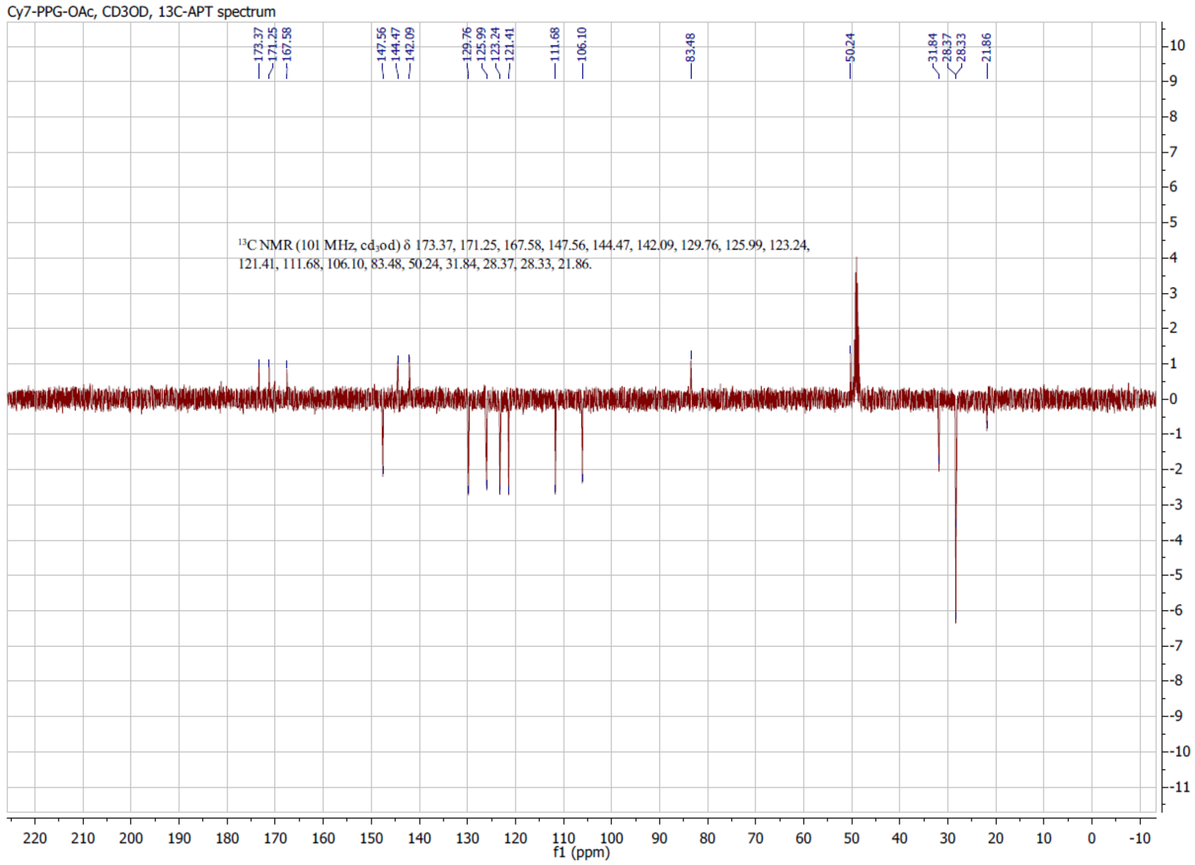

2.4 Cy7-PPG HRMS Spectrum

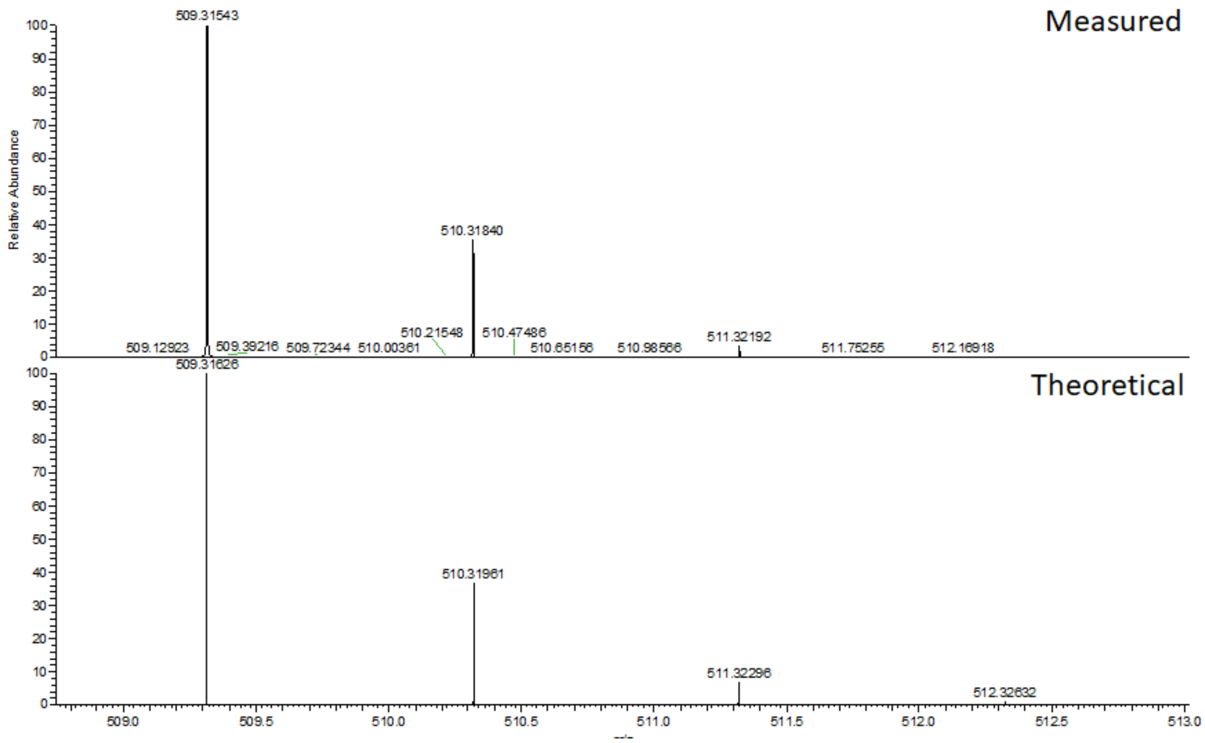

2.5 X-ray Crystal Structures

The single crystals of compounds **ZS-OH** and **ZS-OAc** were grown from room temperature MeCN after supersaturating the solvent with the solute near its boiling point. In all cases, the crystals were kept in the mother liqueur at room temperature. All the single-crystals were mounted on a cryoloop and placed in the nitrogen stream (100 K) of a Bruker-AXS D8 Venture diffractometer. Data collection and processing was carried out using the Bruker APEX3 software suite. A multi-scan absorption correction was applied, based on the intensities of symmetry-related reflections measured at different angular settings (SADABS). The structure was solved using SHELXT and refinement was performed using SHELXL. The hydrogen atoms were generated by geometrical considerations, constrained by idealized geometries and allowed to ride on their carrier atoms with an isotropic displacement parameter related to the equivalent displacement parameter of their carrier atoms. ORTEPs were generated using Mercury 3.5.1 (CCDC) program. If any A- or B-level alerts were raised by CheckCIF a response is given underneath the structure.

ZS-OH

The structure consists of very good diffraction data. However, the acetonitrile solvent molecules in the void could not be reasonably modelled, resulting in a high wR2 and R value. Therefore, the density contribution of the disordered acetonitrile molecules was removed using PLATON_SQUEEZE. The details of the SQUEEZE calculations can be found in the appendix of the CIF file. Nevertheless, the structure was successfully determined and refined.

Figure S3. ORTEP Representation of the Single Crystal of ZS-OH

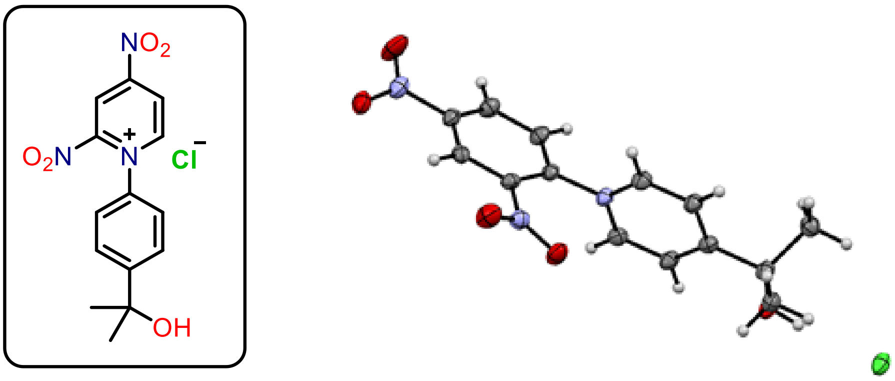

Table S1. Crystal data and structure refinement for ZS-OH

CCDC Identification code	2128353
Empirical formula	$C_{14}H_{14}ClN_3O_5$
Formula weight	339.73
Temperature/K	100.0
Crystal system	orthorhombic
Space group	Pbca
a/Å	17.9506(5)
b/Å	10.7677(3)
c/Å	18.8348(5)
α/°	90
β/°	90
γ/°	90
Volume/Å^3	3640.52(17)
Z	8

ρ_{calc}g/cm^3	1.240
μ/mm^{-1}	2.098
F(000)	1408.0
Crystal size/mm^3	0.1 × 0.08 × 0.05
Radiation	CuKα (λ = 1.54178)
2Θ range for data collection/°	10.67 to 149.644
Index ranges	-22 ≤ h ≤ 22, -13 ≤ k ≤ 9, -23 ≤ l ≤ 23
Reflections collected	26561
Independent reflections	3720 [R_{int} = 0.0657, R_{sigma} = 0.0398]
Data/restraints/parameters	3720/0/215
Goodness-of-fit on F^2	1.084
Final R indexes [I>=2σ (I)]	R_1 = 0.0422, wR_2 = 0.1149
Final R indexes [all data]	R_1 = 0.0510, wR_2 = 0.1204
Largest diff. peak/hole / e Å^{-3}	0.29/-0.29

ZS-OAc

Figure S4. ORTEP Representation of the Single Crystal of ZS-OAc

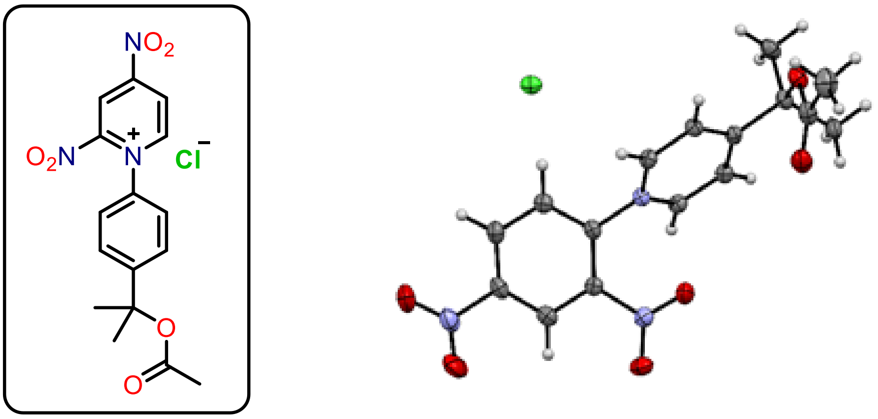

Table S2. Crystal data and structure refinement for ZS-OAc

CCDC Identification code	2128352
Empirical formula	$C_{16}H_{16}ClN_3O_6$
Formula weight	381.77
Temperature/K	100.0
Crystal system	monoclinic
Space group	$P2_1/c$
a/Å	13.1848(4)
b/Å	11.4723(3)
c/Å	11.5868(3)
α/°	90
β/°	91.0590(10)
γ/°	90
Volume/Å^3	1752.32(8)
Z	4
ρ_{calc}g/cm^3	1.447
μ/mm^{-1}	2.290
F(000)	792.0
Crystal size/mm^3	0.198 × 0.181 × 0.071

Radiation	CuKα (λ = 1.54178)
2Θ range for data collection/°	6.704 to 148.892
Index ranges	-16 ≤ h ≤ 16, -14 ≤ k ≤ 14, -11 ≤ l ≤ 14
Reflections collected	20839
Independent reflections	3541 [R_{int} = 0.0610, R_{sigma} = 0.0424]
Data/restraints/parameters	3541/0/239
Goodness-of-fit on F^2	1.086
Final R indexes [I>=2σ (I)]	R_1 = 0.0417, wR_2 = 0.1138
Final R indexes [all data]	R_1 = 0.0468, wR_2 = 0.1187
Largest diff. peak/hole / e Å^{-3}	0.44/-0.29

Alert level B: PLAT430_ALERT_2_B: Short Inter D...A Contact O003..O003. 2.77 Ang, 1-x,1-y,1-z =3_666 Check

Author response: An interaction observed between nitro groups in close proximity.

Figure S4. ORTEP Representation of the Single Crystal of ZS-OAc

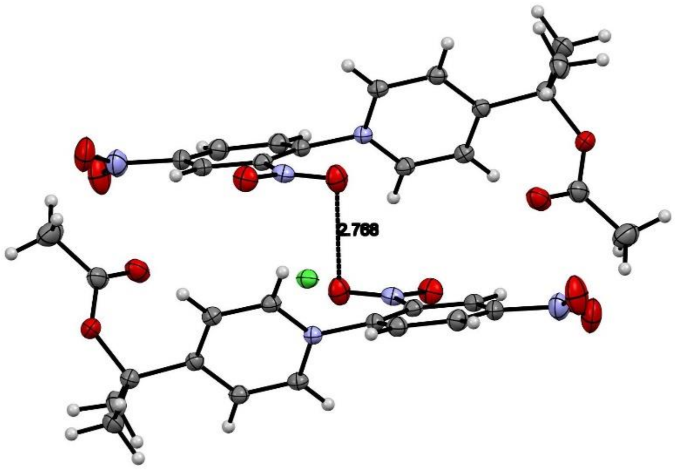

3. Photochemical Methods

3.1 **Cy7-PPG-OAc** Photophysical Properties, Aqueous Solubility and Stability

A solution of **Cy7-PPG-OAc** was prepared (2.0 ml, 1.00 µM in 99:1 milli-Q H_2O/DMSO) in a 3 ml cuvette to test its solubility in this solvent mixture. A UV/Vis spectrum was recorded on an Agilent 8453 UV-visible spectrophotometer at t = 0 min, followed by another at t = 30 min (*Figure S6*). At 30 min **Cy7-PPG-OAc** remains >90% dissolved, showing that very little aggregation or precipitation of the PPG is occurring.

Figure S6. UV/Vis of Cy7-PPG-OAc (1 µM, 99:1 H_2O/DMSO) and stability over time in various biological buffers.

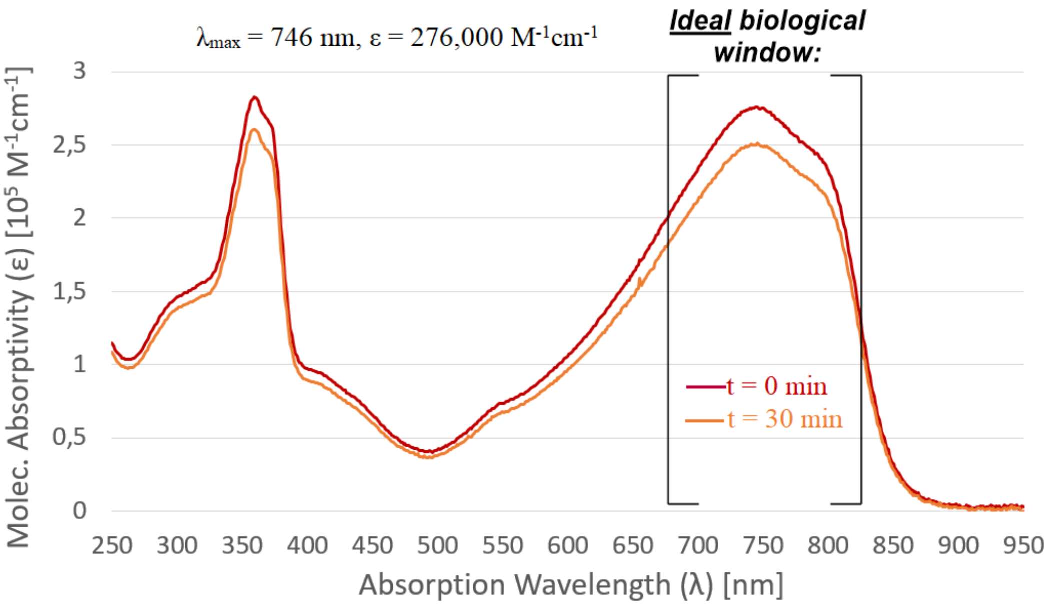

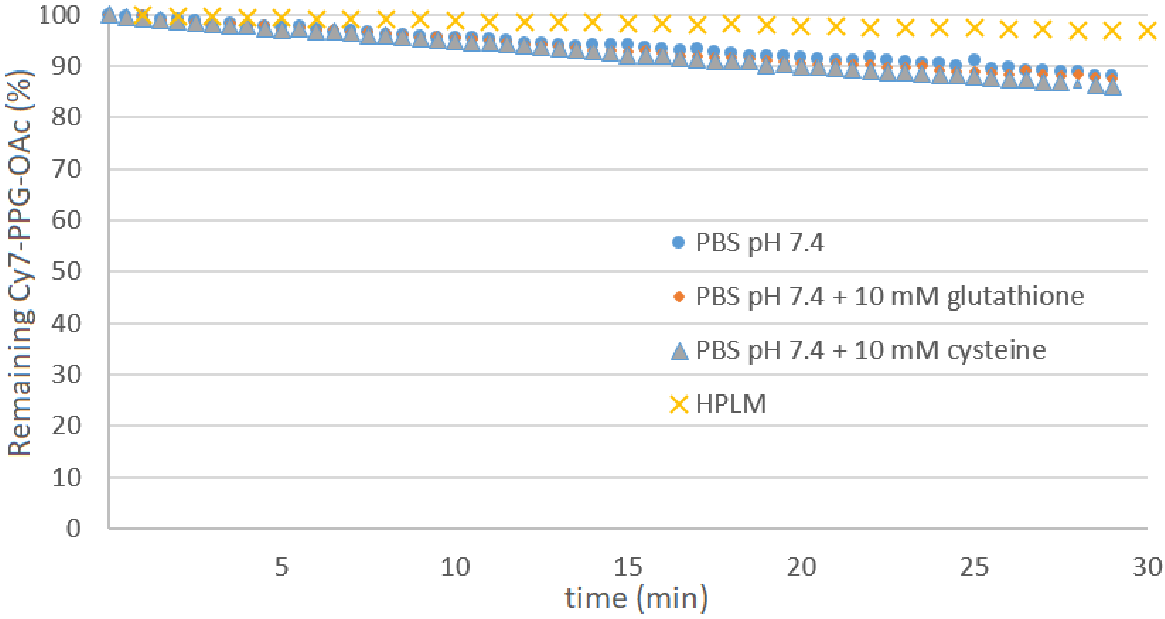

The UV/vis stability of **Cy7-PPG-OAc** was also recorded for solutions prepared in biological buffers: PBS pH 7.4 buffer, PBS pH 7.4 buffer with either glutathione or cysteine at physiologically relevant concentrations (10 mM), and in human plasma-like medium (HPLM, Gibco™ A4899101). Solutions of **Cy7-PPG-OAc** were prepared (2.0 ml, 1.00 µM in 99:1 of a

shown buffer/DMSO) and the 746 nm absorbance was monitored over 30 min. Overall, the reduction in absorbance over 30 min is almost indistinguishable from the slight loss in solubility in 99:1 H_2O/DMSO (*Figure S6*), and thus no adverse effects were observed for the stability of **Cy7-PPG-OAc** in these media, on time scales relevant to the uncaging rate.

The stability of **Cy7-PPG-OAc** solutions in the dark or ambient light was also confirmed by ^1H-NMR. First, an NMR spectrum of **Cy7-PPG-OAc** in 1:1 d_6-DMSO/D_2O was recorded on an Agilent Technologies 400-MR (400/54 Premium Shielded) spectrometer (400 MHz for ^1H nucleus). Then, the NMR samples were left for either 72 h in the dark or for 24 h in ambient laboratory light and a spectrum was recorded again. The comparison of the spectra (*Figure S7)* shows that **Cy7-PPG-OAc** is remarkably stable in ambient light or in the dark (half-life $t_{1/2}$ = days).

Figure S7. NMR Light and Dark Stability of Cy7-PPG-OAc (2 mM, 1:1 D_2O/*d6*-DMSO)

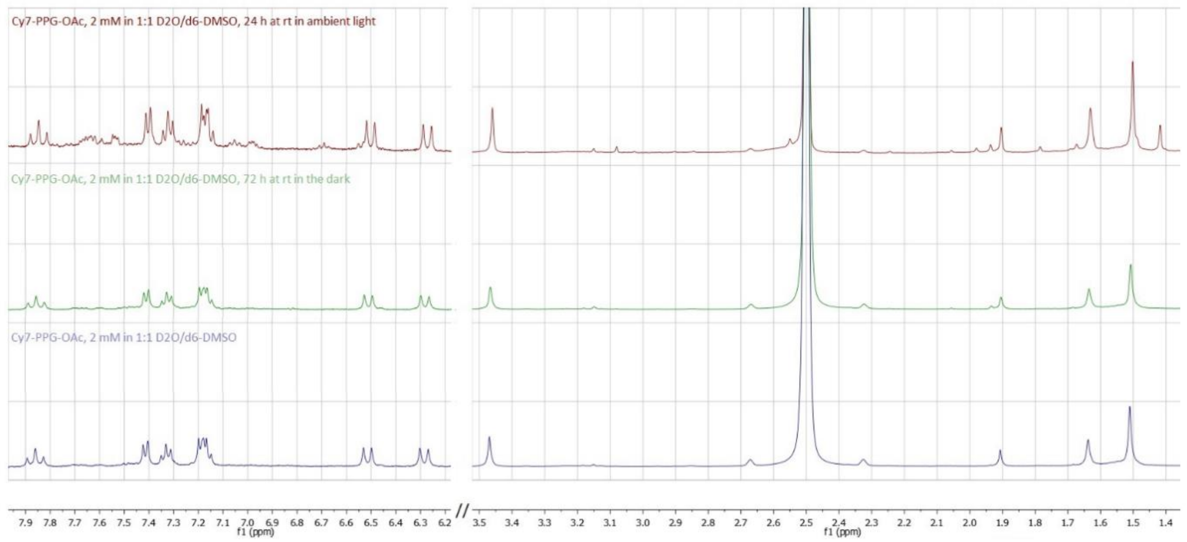

3.2 **Cy7-PPG-OAc** Fluorescence

A solution of **Cy7-PPG-OAc** was prepared (2.0 ml, 10.0 μM in 99:1 milli-Q H_2O/DMSO) in a 3 ml cuvette to measure its emission wavelength and fluorescence quantum yield $\Phi_{fluor.}$. The emission spectrum (*Figure S8*) was recorded on an Edinburgh Instruments FS5 Steady State Spectrofluorometer with integrating sphere.

Figure S8. Excitation and emission spectrum of Cy7-PPG-OAc (10μM, 99:1 H_2O/DMSO)

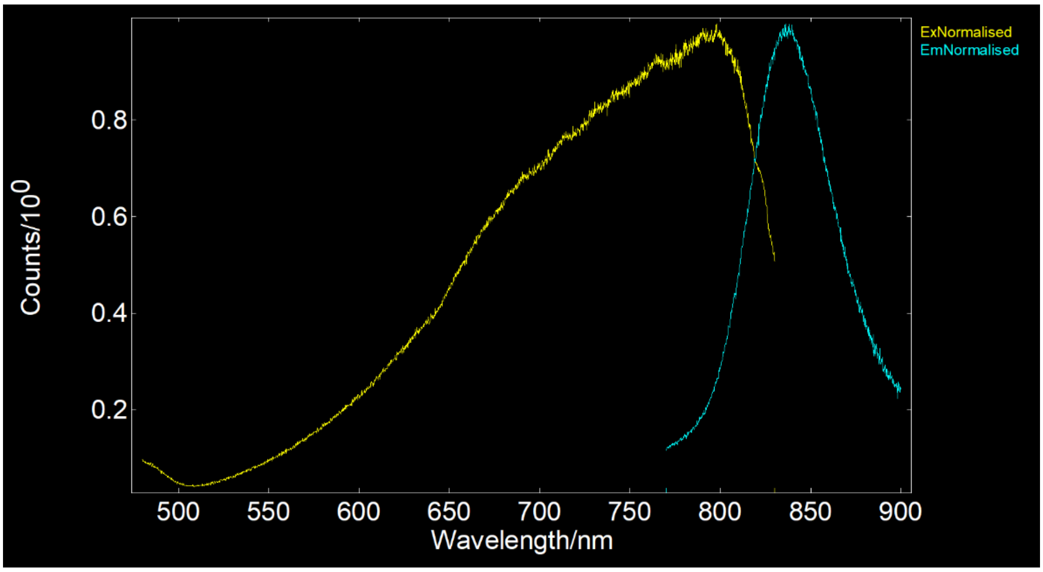

The Φ_{fluor} was measured to be 0.02% over the emission range 827.5 to 876.0 nm, with a subtracted scatter range of 710.5 to 730.5 nm.

3.3 **Cy7-PPG-OAc** Singlet Oxygen Generation

A modified method from a published procedure was used.[5] A 2.0 ml solution containing the photosensitizer to be compared (**Cy7-PPG-OAc** or **Cy7**) (20 µM final concentration) along with 1,3-diphenylisobenzofuran (DPBF) as a singlet oxygen acceptor (100 µM final concentration) in $CHCl_3$ or in 1:9 H_2O/DMSO was placed in a 3 ml cuvette and air-saturated by stirring it for 5 min. Thereafter, the photosensitizer + DPBF solutions were irradiated at the main absorbance band of **Cy7-PPG-OAc** (λ_{max} = 746 nm, absorbance > 2 at 20 µM) or **Cy7** (λ_{max} = 736 nm, absorbance > 2 at 20 µM) with a UV/Vis-mounted Sahlmann Photochemical Solutions 760 nm LED system (3 x 350 mW, peak wavelength = 761 nm, FWHM 18 nm). During 760 nm irradiation, the solutions' UV/Vis spectrum was monitored for the disappearance of the main absorption band of DPDF at 445 nm, signifying consumption of the singlet oxygen acceptor by the generated singlet oxygen, to produce the graph shown below (*Figure 9*).

Figure S9. Consumption of DPBF (100 µM) over time during the 760 nm irradiation in the presence of Cy7-PPG-OAc (20 µM) or Cy7 (20 µM)

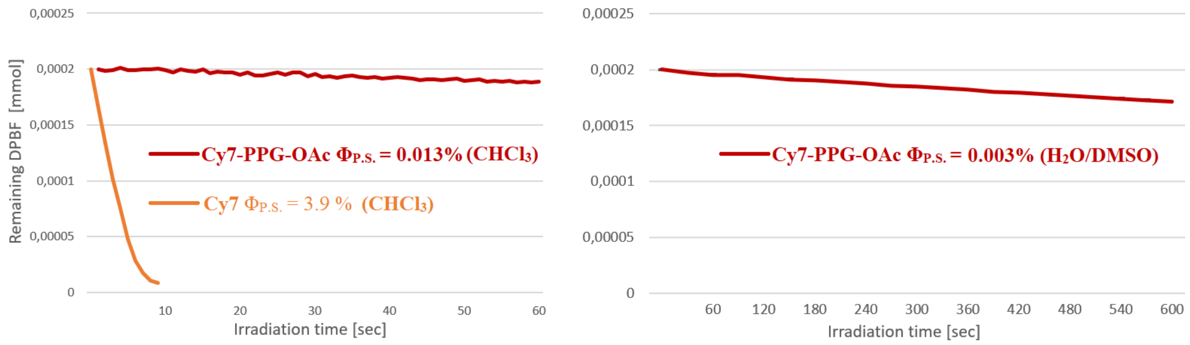

$$\text{Cy7-PPG-OAc } \Phi_{P.S.} = \frac{A_{445nm} \text{ Cy7-PPG slope}}{A_{445nm} \text{ Cy7 slope}} \times \text{Cy7 } \Phi_{P.S.}$$

The slope of the consumption of DPBF from the irradiation of **Cy7-PPG-OAc** was divided by to the slope of the consumption of DPBF from the irradiation of **Cy7**, and finally multiplied by

the photosensitization quantum yield of **Cy7** ($\Phi_{P.S.}$ = 3.9%, $CHCl_3$, 760 nm irradiation),[5] according to this equation:

$$\textbf{Cy7-PPG-OAc}\ \Phi_{P.S.} = \frac{A_{445nm}\ \text{Cy7-PPG slope}}{A_{445nm}\ \text{Cy7 slope}} \times \textbf{Cy7}\ \Phi_{P.S.}$$

Thus, **Cy7-PPG-OAc** was found to generate only small amounts of singlet oxygen, with a quantum yield $\Phi_{P.S.}$ of 0.013±0.0004% ($CHCl_3$) or 0.003±0.0001% (1:9 H_2O/DMSO).

3.4 Photoheterolysis of **Cy7-PPG-OAc**

The λ = 760 nm light-induced photoheterolysis of **Cy7-PPG-OAc** was monitored by UV/vis in triplicate. A solution of **Cy7-PPG-OAc** was prepared (3.0 ml, 1.00 μM in degassed 99:1 milli-Q H_2O/DMSO) in a 3 ml cuvette. A UV/Vis spectrum was recorded on an Agilent 8453 UV-visible spectrophotometer at t = 0 min, and the cuvette was removed then irradiated with a Sahlmann Photochemical Solutions 760 nm LED system (3 x 350 mW, peak wavelength = 761 nm, FWHM 18 nm) and a new UV/Vis spectrum was recorded at various time intervals until the main absorption band at 746 nm had decreased by ~90%. The averaged spectra for the various time points were overlaid and are shown below (*Figure S10a*).

Figure S10. AcOH photoheterolysis proposed mechanism[6] and monitoring by UV/vis

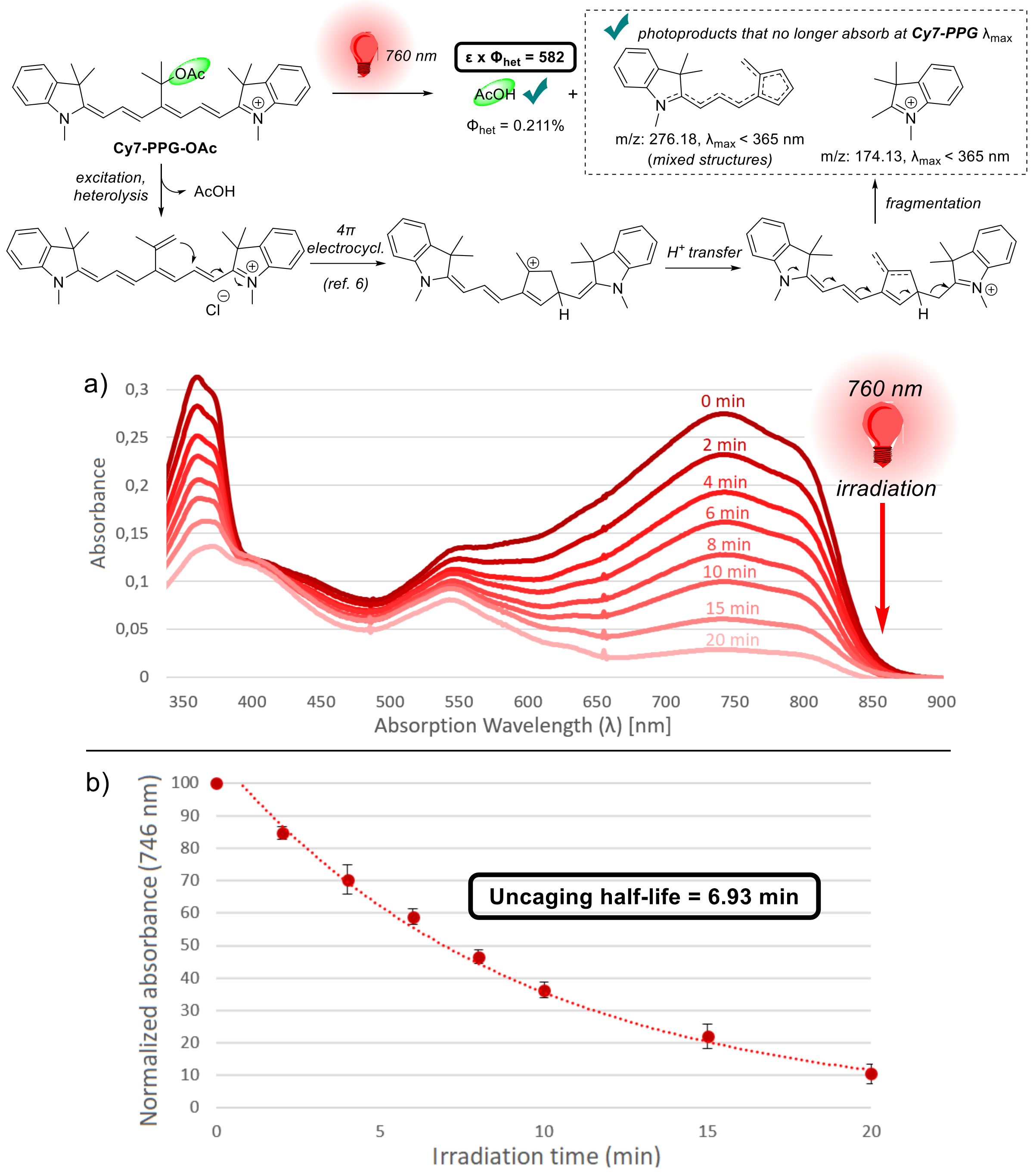

The absorbance at 746 nm was then normalized and plotted versus time, to generate the graph shown above (*Figure S10b*). Exponential fitting ($R^2 = 0.992$) resulted in a calculated heterolysis half-life $t_{1/2} = 6.93 \pm 0.18$ min.

The accurate quantitative release of the payload AcOH from **Cy7-PPG-OAc** (0.5 ml, 2.0 mM in 1:1 d_6-DMSO/D_2O, non-degassed) by 760 nm irradiation was monitored by ^{1}H-NMR. At 2.0 mM concentration, **Cy7-PPG-OAc** exhibits an optical absorbance of >2 at 760 nm, meaning that >99% of photons of this wavelength to be passed through the **Cy7-PPG-OAc** samples would be absorbed. NB: during these conditions (1000 nmol PPG) one expects much longer uncaging times, as the overall uncaging rate depends solely on the photon flux of the light source and on the heterolysis quantum yield Φ_{het} of the PPG (i.e. the overall uncaging rate is zeroth-order in PPG concentration).

The **Cy7-PPG-OAc** samples in tissue were then mounted on an NMR-tube holder and irradiated with a Sahlmann Photochemical Solutions 760 nm LED system (3 x 350 mW, peak wavelength = 761 nm, FWHM 18 nm). At 1 h intervals, the NMR tube was removed from the NMR-tube holder and an NMR spectrum was recorded on an Agilent Technologies 400-MR (400/54 Premium Shielded) spectrometer (400 MHz for ^{1}H nucleus) to monitor the AcOH released (*Figure S11)*. The correct peak for AcOH was identified by adding a small amount of commercial AcOH dissolved in d_6-DMSO at either the beginning or at the end of the irradiation experiment, confirming that the signal whose accumulation was being monitored was that of AcOH.

Figure S11. NMR photoheterolysis of AcOH from Cy7-PPG-OAc over time

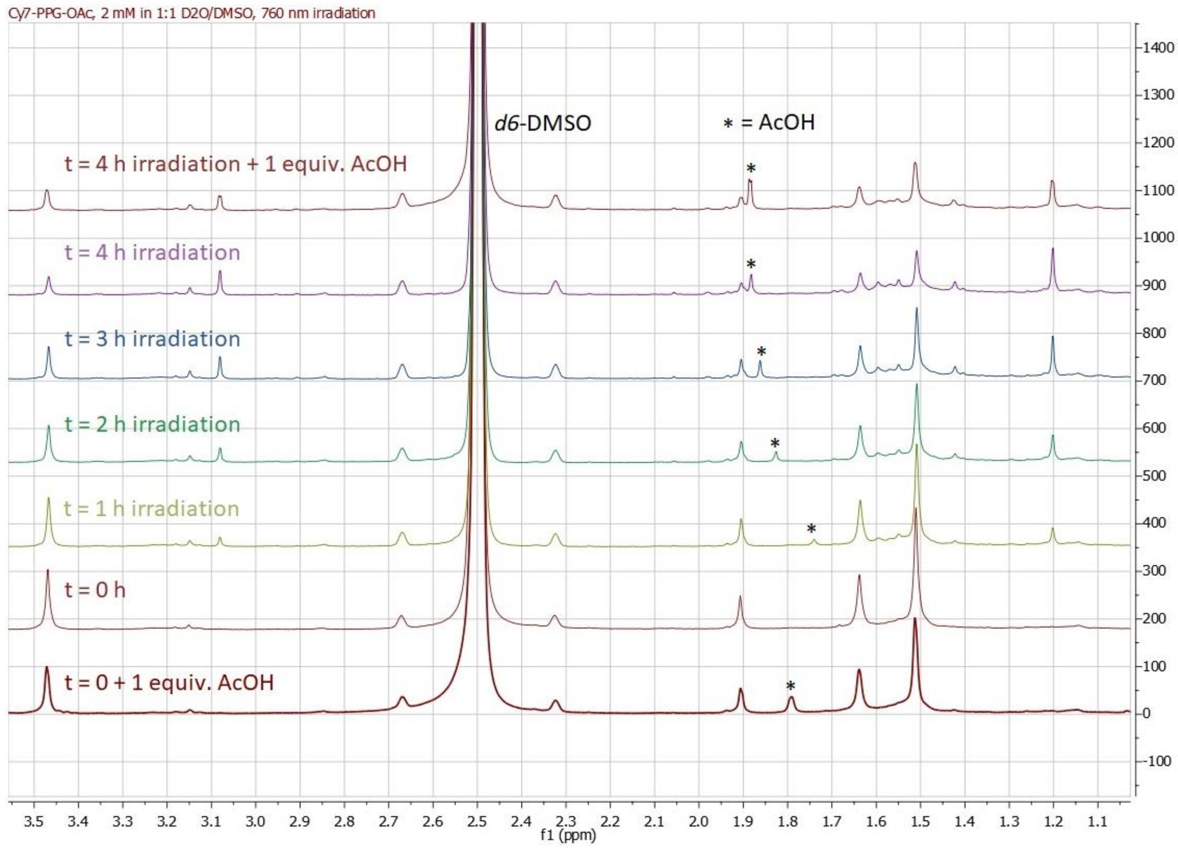

3.5 Photoheterolysis Quantum Yield of **Cy7-PPG-OAc**

A modified procedure from the literature was used.[7] The photoheterolysis quantum yield $\Phi_{het.}$ For **Cy7-PPG-Oac** was determined by conducting a photoheterolysis NMR experiment in parallel with a suitable actinometer. For this purpose, **BODIPY-PPG-OAc** (Φ_{het} = 0.099%, in MeOH), a PPG with a known quantum yield Φ_{het}, that uncages an identical payload (AcOH) was selected as the actinometer. The release of the payload AcOH from separate 0.5 ml 1.0 mM solutions of **Cy7-PPG-OAc** (in 9:1 d_6-DMSO/D_2O, non-degassed) and the actinometer (in MeOD) was monitored by ^{1}H-NMR. The solvent was pre-treated with a small amount of acetone to serve as the internal standard for the AcOH release quantification. At 1.0 mM concentration, both **Cy7-PPG-OAc** and the actinometer exhibit optical absorbances of >2 at 530 nm, meaning that >99% of photons of this wavelength to be passed through the samples would be absorbed. The **Cy7-PPG-OAc** and the actinometer samples were then irradiated side-by-side with a Sahlmann Photochemical Solutions 530 nm LED system (3 x 270 mW, peak wavelength = 526 nm, FWHM 35.1 nm). At 15 min intervals an NMR spectrum was recorded on an Agilent Technologies 400-MR (400/54 Premium Shielded) spectrometer (400 MHz for ^{1}H nucleus) to quantify the AcOH released. The correct peak for AcOH was identified by adding a small amount of commercial AcOH dissolved in d_6-DMSO at the end of the experiment, confirming that the peak whose accumulation was being monitored was the correct one. The irradiation experiments were conducted in triplicate, and the generated AcOH was averaged, normalized, and plotted versus time for both the **Cy7-PPG-OAc** and the actinometer samples, to produce the graph below (*Figure S12*).

Figure S12. Quantified photoheterolysis of AcOH from Cy7-PPG-OAc or BODIPY-PPG-OAc over time

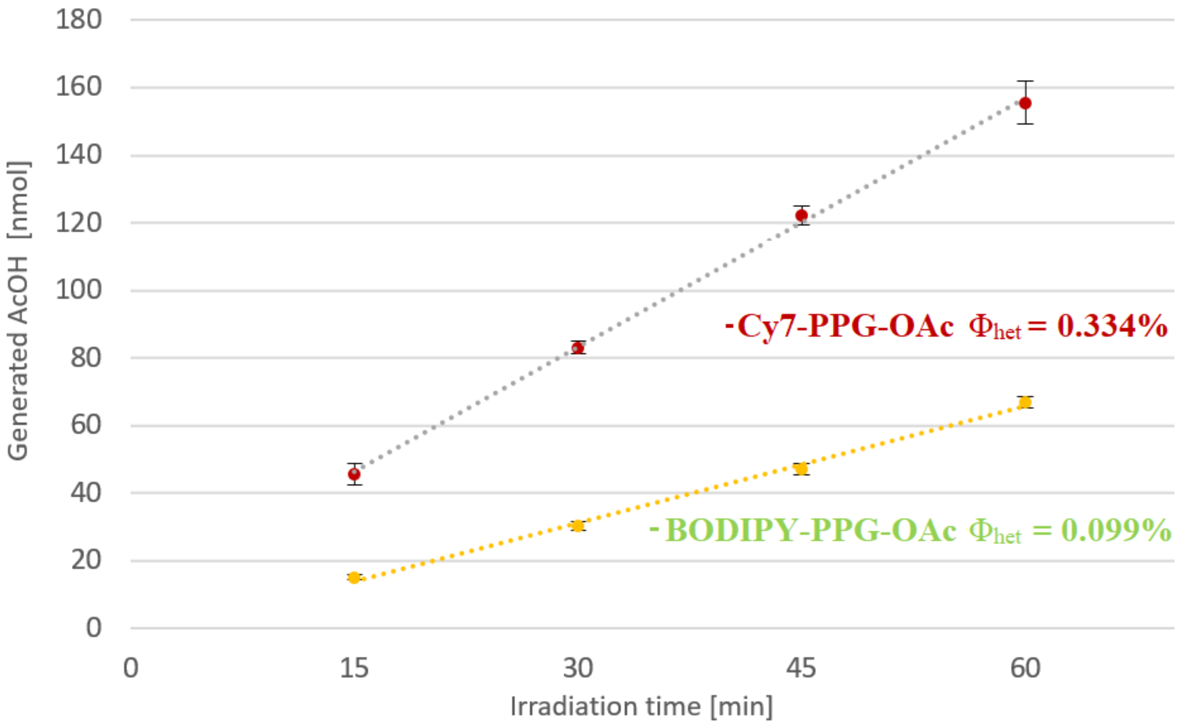

$$\text{Cy7-PPG-OAc } \Phi_{het} = \frac{\text{AcOH Cy7-PPG slope}}{\text{AcOH BODIPY-PPG slope}} \times \text{BODIPY-PPG-OAc } \Phi_{het}$$

The slope of the generation of AcOH from the irradiation of **Cy7-PPG-OAc** was divided by the slope of the generation of AcOH from the irradiation of actinometer **BODIPY-PPG-OAc**, and finally multiplied by the Φ_{het} of **BODIPY-PPG-OAc**,[7] according to this equation:

$$\textbf{Cy7-PPG-OAc}\ \Phi_{het} = \frac{\text{AcOH Cy7-PPG slope}}{\text{AcOH BODIPY-PPG slope}} \times \textbf{BODIPY-PPG-OAc}\ \Phi_{het}$$

Thus, **Cy7-PPG-OAc** was found to release AcOH with a quantum yield Φ_{het} of 0.334 (±0.014% standard error).

<u>3.6 Photoheterolysis of **Cy7-PPG-OAc** in tissue</u>

The accurate quantitative release of the payload AcOH from **Cy7-PPG-OAc** (0.5 ml, 2.0 mM in 1:1 d_6-DMSO/D_2O, 1000 nmol total) by 760 nm irradiation through various tissue samples was monitored by ^{1}H-NMR. The d_6-DMSO solvent was pre-treated with a small amount of acetone to serve as the internal standard for the AcOH release quantification. At 2.0 mM concentration, **Cy7-PPG-OAc** exhibits an optical absorbance of >2 at 760 nm, meaning that >99% of photons of this wavelength to be passed through the **Cy7-PPG-OAc** samples would be absorbed. NB: during these conditions (1000 nmol PPG) one expects much longer uncaging times, as the overall uncaging rate depends solely on the photon flux of the light source and on the heterolysis quantum yield Φ_{het} of the PPG (i.e. the overall uncaging rate is zeroth-order in PPG concentration).

The tissue samples *Hollandse Nieuwe* (raw herring, Vishandel Zwier, Groningen Market) and *Speklap* (raw pork belly, Slager Wilner, Groningen Market) were cut into cuboid phantoms with a length and width of approximately 1 cm. An incision was made into these cuboid samples such that an NMR tube containing the 2.0 mM solutions of **Cy7-PPG-OAc** in the deuterated solvent system could be inserted along the longest dimension of the cuboid (i.e. the NMR tubes were surrounded by roughly 0.5 cm of tissue). The **Cy7-PPG-OAc** samples in tissue were then mounted on an NMR-tube holder and irradiated with a Sahlmann Photochemical Solutions 760 nm LED system (3 x 350 mW, peak wavelength = 761 nm, FWHM 18 nm) directly through the 0.5 cm of tissue. At 1 h intervals the NMR tube was removed from the tissue and an NMR spectrum was recorded on an Agilent Technologies 400-MR (400/54 Premium Shielded) spectrometer (400 MHz for ^{1}H nucleus) to quantify the AcOH released. The correct signal for AcOH was identified by adding a small amount of commercial AcOH dissolved in d_6-DMSO at the end of the experiment, confirming that the signal whose accumulation was being monitored was the correct one. The generated AcOH from the irradiation experiments was plotted versus time, to produce the graphs below (*Figure 13*).

Figure S13. Cy7-PPG-OAc payload uncaging with 760 nm light inside tissue phantoms

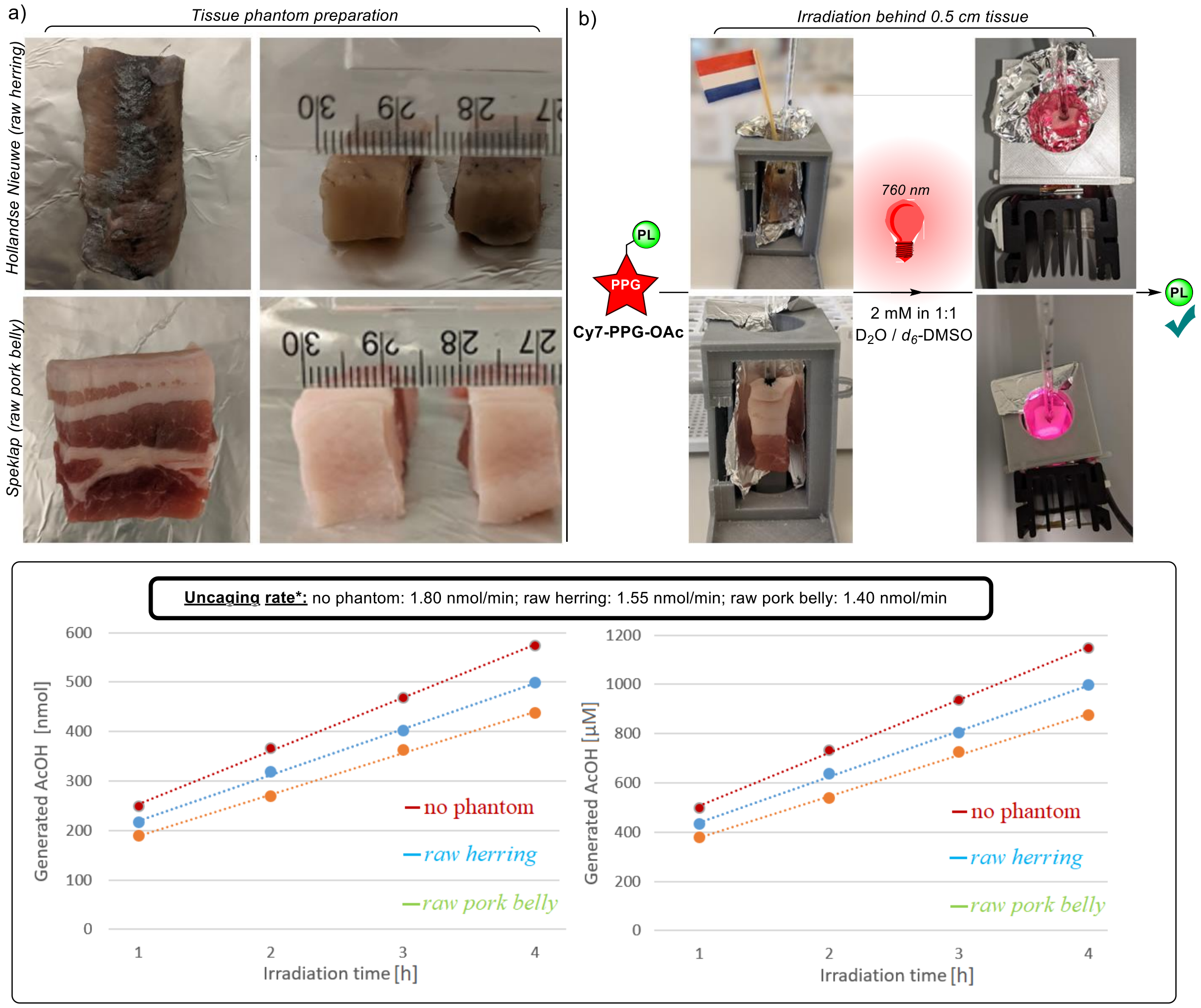

4. References

[1] H. S. Yu, X. He, S. L. Li, D. G. Truhlar, *Chem. Sci.* **2016**, *7*, 5032–5051.

[2] J. Zheng, X. Xu, D. G. Truhlar, *Theor. Chem. Acc.* **2011**, *128*, 295–305.

[3] A. V. Marenich, C. J. Cramer, D. G. Truhlar, *J. Phys. Chem. B* **2009**, *113*, 6378–6396.

[4] L. Štacková, P. Štacko, P. Klán, *J. Am. Chem. Soc.* **2019**, *141*, 7155–7162.

[5] H.-J. Adick, R. Schmidt, H.-D. Brauer, *J. Photochem. Photobiol. A Chem.* **1990**, *54*, 27–30.

[6] G. Alachouzos, A. J. Frontier, *Angew. Chemie Int. Ed.* **2017**, *56*, 15030–15034.

[7] P. Shrestha, K. C. Dissanayake, E. J. Gehrmann, C. S. Wijesooriya, A. Mukhopadhyay, E. A. Smith, A. H. Winter, *J. Am. Chem. Soc.* **2020**, *142*, 15505–15512.
